# Ethnic Differences in the Prevalence of Mental Health Disorders Among Individuals with Diabetes: A Systematic Review and Meta-analysis

**DOI:** 10.1007/s40615-025-02656-7

**Published:** 2025-11-10

**Authors:** Ngozika Esther Ezinne, Kingsley Emwinyore Agho, Aunty Kerrie Doyle, Minha Lee, Sia Jung, Uchechukwu Levi Osuagwu

**Affiliations:** 1 Bathurst Rural Clinical School, School of Medicine, Western Sydney University, Bathurst, 2795 NSW Australia; 2 Translational Health Research Institute, Western Sydney University, Campbelltown, 2560 NSW Australia; 3 Optometry Unit, Department of Clinical Surgical Sciences, Faculty of Medical Science, University of the West Indies, Saint Augustine Campus, Trinidad, Trinidad and Tobago; 4 School of Health Sciences, Western Sydney University, Campbelltown, 2560 Australia

**Keywords:** Diabetes, Ethnicity, Depression, Anxiety, Post-traumatic stress disorder, Schizophrenia

## Abstract

**Aim:**

This systematic review and meta-analysis aimed to estimate the prevalence of mental health disorders among adults (≥ 18 years) with diabetes in different ethnic populations.

**Methods:**

A comprehensive search was conducted in five databases (EMBASE, Medline, PsycINFO, Scopus, and CINAHL) for peer-reviewed observational studies published in English between 2014 and 2024. Studies reporting the prevalence of mental health disorders determined using validated self-report screening tools or clinical diagnoses were included. Meta-analyses using random-effects models were conducted. Subgroup analyses and meta-regressions explored differences by ethnicity, diabetes type, diagnostic method, duration of diabetes, country income level, and indigenous status.

**Results:**

Of 17,723 identified records, 53 studies met the inclusion criteria. The pooled prevalence of mental health disorders among adults with diabetes was 25.78% (95% CI: 23.27–28.29), with high heterogeneity (I^2^ = 99.9%). For those with known ethnic group, the prevalence of mental health disorder was highest in North Africa and the Middle East (32.44%, 95% CI: 27.71–37.45), followed by Hispanics or Latinos (28.36%, 95% CI: 25.20–31.57) and lowest prevalence was among Oceanians (10.42%). Post traumatic stress disorder (PTSD) (37.79%) and depression (27.55%) were the most common mental health disorders, while schizophrenia was the least prevalent (3.45%). Type 2 diabetes (31.91%), diabetes duration < 10 years (26.21%), low-income countries (40.63%), and use of non self-report tools (29.19%) were associated with higher prevalence of mental health disorder. Non-Indigenous populations also had a greater burden (26.45%) than Indigenous groups (17.65%).

**Conclusion:**

Approximately one in four adults with diabetes experiences mental health disorders, with significant variation by ethnicity, diabetes type, diagnostic method, and socioeconomic context. Culturally tailored, integrated care approaches are needed to address disparities in mental health among people with diabetes.

**Supplementary Information:**

The online version contains supplementary material available at https://doi.org/10.1007/s40615-025-02656-7.

## Introduction

Diabetes mellitus (DM) is a chronic metabolic disorder characterized by persistent hyperglycemia resulting from insufficient insulin production or the body’s inability to use insulin effectively [1, 2]. It represents a growing global public health concern, affecting approximately 422 million individuals worldwide—including 10.5% of adults aged 20–79 years in high-income countries [3]. An additional 240 million people are estimated to have undiagnosed diabetes, with nearly half of those affected unaware of their condition [1, 3]. The Western Pacific region, followed by Asia, North Africa, and the Middle East, bears the highest prevalence, with global healthcare expenditures related to Diabetes reaching USD 966 billion—a 316% increase over the past 15 years [3]. Diabetes is also a major contributor to morbidity and mortality, responsible for approximately 6.7 million deaths annually, and is associated with complications such as amputations, vision loss, renal failure, and cardiovascular diseases [1, 4].

In addition to its physical health consequences, diabetes is closely linked to a range of mental health disorders, including depression, anxiety, post-traumatic stress disorder (PTSD), and schizophrenia [5, 6]. Mental health disorders are highly prevalent among individuals with chronic illnesses and substantially impact quality of life and treatment outcomes [5, 7]. Globally, an estimated 970 million people are affected by mental health conditions, with depression and anxiety impacting approximately 280 million and 301 million individuals, respectively [8, 9]. These disorders impair emotional regulation and behavioral functioning, contributing to decreased productivity and overall well-being [4]. Individuals with diabetes who also experience mental health disorders face additional challenges in adhering to treatment regimens, maintaining healthy lifestyles, and managing glycemic control [10–12].

Depression alone is diagnosed in approximately 10% of individuals with Diabetes, with up to 25% reporting significant depressive symptoms [13]. Reported prevalence rates of mental health disorders among individuals with diabetes and prediabetes vary widely—from 1.26% to 50%—depending on the study population and setting [4, 12, 14–17]. For instance, Farooqi et al. [17] observed higher depression rates among individuals with diabetes compared to those without the condition, while Chien and Lin et al. [14] reported nearly double the prevalence of mental health disorders in Taiwanese individuals with diabetes compared to the general population. Dziewa et al. [16] also found a higher prevalence of anorexia nervosa and eating disorders among females with type 2 diabetes in Poland.

The relationship between diabetes and mental health is complex and bidirectional. Psychological distress may increase the risk of developing diabetes through behavioral and physiological pathways, including increased stress hormones, inflammation, and unhealthy lifestyle behaviors such as poor diet and physical inactivity [11, 18]. Despite increasing recognition of this interplay, the mechanisms underlying these associations remain insufficiently understood and require further investigation. Conversely, the daily burden of diabetes self-management—including medication adherence, glucose monitoring, and fear of complications—can contribute to or exacerbate mental health conditions such as depression, anxiety, and PTSD [13, 19]. Mental health disorders can in turn impair self-care behaviors, reduce treatment adherence, and worsen glycemic control, further complicating diabetes management. Despite increasing recognition of this interplay, the underlying mechanisms remain insufficiently understood and require further investigation.

Ethnicity was identified as a key factor influencing the prevalence of both diabetes and mental health disorders. Certain ethnic groups, including Black, Indigenous, and Hispanic populations, are disproportionately affected by both conditions due to systemic factors such as socioeconomic disadvantage, healthcare access disparities, and cultural stigma. For example, African American and American Indian populations have been shown to experience higher rates of post-traumatic stress disorder (PTSD), often linked to historical trauma and ongoing structural inequities [15, 20]. PTSD may be underrecognized in diabetes research despite its physiological consequences, such as increased inflammation and disrupted metabolic control, which may exacerbate diabetes outcomes. Similarly, ethnic differences in depression and anxiety have been widely reported. Aujla et a [18] found that depression prevalence was higher among Caucasians than African Americans (32% vs. 23%), while Chaturvedi et al. [21] reported higher anxiety levels in populations from Ukraine, Saudi Arabia, and Argentina. Tegegne et al. [9] documented high depression rates in Ethiopia, while Tanaka et al. [22] observed lower rates in Japan.

Despite recognizing these disparities, there is a lack of studies providing pooled estimates of the global prevalence of mental health disorders among individuals with diabetes, highlighting the need for systematic reviews and meta-analyses. This study therefore aims to systematically review and estimate the pooled prevalence of mental health disorders among individuals with diabetes across various ethnic groups globally. By doing so, we seek to better understand ethnic differences in the prevalence of mental health disorders and identify regions where targeted interventions are most needed.

## Materials and Methods

### Study Design

This systematic review and meta-analysis followed the Preferred Reporting Items for Systematic Reviews and Meta-Analyses (PRISMA) guidelines. The protocol was registered in the International Prospective Register of Systematic Reviews (PROSPERO) under registration number CRD42024617719 on 22 November 2024.

### Search Strategies

A comprehensive search was conducted in five databases—MEDLINE, Embase, CINAHL (EBSCO), APA PsycInfo (EBSCO), and Scopus—between November 13, 2024, and December 17, 2024. The search strategy combined keywords and subject headings based on the PEO (Population, Exposure, Outcome) framework, covering the following concepts: (1) diabetes or prediabetes, (2) adults, (3) mental health disorders, and (4) ethnicity. The full search strategies for the different databases used in this study are shown in Table 1. Additionally, manual reference checking of included articles and relevant reviews was conducted. Search results were managed using Covidence (version 2024) for duplicate removal, screening, and full-text retrieval.

**Table 1:** Full search strategy for the different databases

Database searched	Terms used	Date searched
Medline	((diabetes mellitus/or diabetes mellitus, experimental/or Diabetes mellitus, type 1/or Diabetes mellitus, type 2/or diabetes mellitus, lipoatrophic/or latent autoimmune diabetes in adults/or prediabetic state/) OR (diabet* or prediabet* or “impaired glucose tolerance” or “impaired fasting glucose” or T2D* or T1D*).ti,ab.) AND (exp Adult/OR (adult* or middle aged or aged or person* or people or patient*).ti,ab.) AND ((mental disorders/or exp anxiety disorders/or exp dissociative disorders/or exp "feeding and eating disorders"/or exp mood disorders/or exp paraphilic disorders/or exp personality disorders/or exp "schizophrenia spectrum and other psychotic disorders"/or exp "trauma and stressor related disorders"/or exp Mental Health/or depersonalization/or depression/) OR (“mental illness” or “mental health disorder” or “mental problem” or “mental disorder” or “mental disease” or psychiatric or “psychotic disorder” or psychosis or psychotrauma or “mood disorder” or schizophrenia or “delusional disorder” or depressi* or “bipolar disorder” or anxiety or “panic disorder” or “obsessive–compulsive disorder” or “post-traumatic stress disorder” or “eating disorder” or “anorexia nervosa” or “bulimia nervosa” or “binge-eating disorder” or “personality disorder”).ti,ab.) AND ((exp "health disparate minority and vulnerable populations"/or exp population groups/) OR (ethnic* or racial* or race or cultur* or minority* or immigrant* or migrant* or native or Indigenous or Aboriginal or “First Nation*” or “African American” or “American Indian” or “Mexican American” or Caucasian or Maori or Hispanic or Latino or Caribbean or “Middle Eastern” or Chinese).ti,ab.) AND yr = ”2014–2024”	13/11/2024
EMBASE	((exp diabetes mellitus/or exp impaired glucose tolerance/or exp diabetic patient) OR (diabet* or prediabet* or “impaired glucose tolerance” or “impaired fasting glucose” or T1D* or T2D*).ti,ab.) AND ((adult/or exp aged/or exp middle aged/or exp young adult/) OR (adult* or middle aged or aged or person* or people or patient*).ti,ab.) AND (exp mental health/or mental disease/or exp anxiety disorder/or behavior disorder/or exp mood disorder/or exp personality disorder/or exp psychosis/or exp schizophrenia spectrum disorder/) OR (“mental illness” or “mental health disorder” or “mental problem” or “mental disorder” or psychiatric or “psychotic disorder” or psychosis or “mood disorder” or schizophrenia or “delusional disorder” or depressi* or “bipolar disorder” or anxiety or “panic disorder” or “obsessive–compulsive disorder” or “post-traumatic stress disorder” or “eating disorder” or “anorexia nervosa” or “bulimia nervosa” or “binge-eating disorder” or “personality disorder”).ti,ab.) AND (exp ethnicity/or exp ethnic group/or exp minority group/) OR (ethnic* or racial* or race or cultur* or minority* or immigrant* or migrant* or native or Indigenous or Aboriginal or “First Nation*” or “African American” or “American Indian” or “Mexican American” or Caucasian or Maori or Hispanic or Latino or Caribbean or “Middle Eastern” or Chinese).ti,ab.) AND yr = ”2014–2024”	20/11/2024
APA PsycInfo	((DE "Diabetes Mellitus" OR DE "Type 1 Diabetes" OR DE "Type 2 Diabetes" OR DE "Type 1 Diabetes" OR DE "Diabetes") OR (diabet* or prediabet* or “impaired glucose tolerance” or “impaired fasting glucose” or T2D* or T1D*).ti,ab.) AND (AG "adulthood (18 yrs & older)") or (AG "aged (65 yrs & older)") or (AG "middle age (40–64 yrs)") or (AG "thirties (30–39 yrs)") or (AG "very old (85 yrs & older)") or (AG "young adulthood (18–29 yrs)") OR (TI (adult* or middle aged or aged or person* or people or patient*) OR AB (adult* or middle aged or aged or person* or people or patient*)) AND ((DE "Affective Disorders" OR DE "Major Depression" OR DE "Persistent Depressive Disorder" OR DE "Generalized Anxiety Disorder" OR DE "Panic Disorder" OR DE "Bipolar I Disorder" OR DE "Bipolar II Disorder" OR DE "Chronic Mental Illness" OR DE "Chronic Psychosis" OR DE "Anorexia Nervosa" OR DE "Binge Eating Disorder" OR DE "Bulimia" OR DE "Feeding Disorders" OR DE "Obsessive Compulsive Disorder" OR DE "Psychosis" OR DE "Serious Mental Illness" OR DE "Acute Stress Disorder" OR DE "Posttraumatic Stress Disorder" OR DE "Complex PTSD" OR DE "Mental Health Disparities") OR (TI (“mental illness” or “mental health disorder” or “mental problem” or “mental disorder” or “mental disease” or psychiatric or “psychotic disorder” or psychosis or psychotrauma or “mood disorder” or schizophrenia or “delusional disorder” or depressi* or “bipolar disorder” or anxiety or “panic disorder” or “obsessive–compulsive disorder” or “post-traumatic stress disorder” or “eating disorder” or “anorexia nervosa” or “bulimia nervosa” or “binge-eating disorder” or “personality disorder”).ti,ab.) AND ((DE "Racial and Ethnic Groups" OR DE "African Cultural Groups" OR DE "Asians" OR DE "Black People" OR DE "European Cultural Groups" OR DE "Indigenous Populations" OR DE "Latinos/Latinas" OR DE "Middle Eastern and North African Cultural Groups" OR DE "Multiracial" OR DE "North American Cultural Groups" OR DE "Oceanian Cultural Groups" OR DE "People of Color" OR DE "Romanies" OR DE "South American Cultural Groups" OR DE "White People" OR DE "Majority Groups" OR DE "Marginalized Groups" OR DE "Minority Groups") OR (ethnic* or racial* or race or cultur* or minority* or immigrant* or migrant* or native or Indigenous or Aboriginal or “First Nation*” or “African American” or “American Indian” or “Mexican American” or Black or White or Caucasian or European or Asian or Maori or Hispanic or Latino or Caribbean or “Middle Eastern” or Chinese).ti,ab.) AND (Year: “2014–2024”)	28/11/2024
CINAHL	((MH "Diabetes Mellitus") OR (MH "Diabetes Mellitus, Type 1 + ") OR (MH "Diabetes Mellitus, Type 2") OR (MH "Prediabetic State")) OR (diabet* or prediabet* or “impaired glucose tolerance” or “impaired fasting glucose” or T1D* or T2D*).ti,ab.) AND (((MH "Adult") OR (MH "Aged") OR (MH "Middle Age") OR (MH "Young Adult")) OR (adult* or middle aged or aged or person* or people or patient*).ti,ab.) AND (((MH "Mental Disorders") OR (MH "Mental Disorders, Chronic") OR (MH "Depression") OR (MH "Generalized Anxiety Disorder") OR (MH "Obsessive–Compulsive Disorder") OR (MH "Panic Disorder") OR (MH "Stress Disorders, Post-Traumatic") OR (MH "Affective Disorders") OR (MH "Anxiety Disorders") OR (MH "Affective Disorders, Psychotic") OR (MH "Psychotic Disorders") OR (MH "Bipolar Disorder") OR (MH "Schizophrenia") OR (MH "Schizoaffective Disorder") OR (MH "Personality Disorders") OR (MH "Paraphilic Disorders") OR (MH "Neurotic Disorders")) OR (“mental illness” or “mental health disorder” or “mental problem” or “mental disorder” or “mental disease” or psychiatric or “psychotic disorder” or psychosis or psychotrauma or “mood disorder” or schizophrenia or “delusional disorder” or depressi* or “bipolar disorder” or anxiety or “panic disorder” or “obsessive–compulsive disorder” or “post-traumatic stress disorder” or “eating disorder” or “anorexia nervosa” or “bulimia nervosa” or “binge-eating disorder” or “personality disorder”).ti,ab.) AND (((MH "Ethnic Groups + ") OR (MH "Minority Groups") OR (MH "Migrants") OR (MH "Immigrants") OR (MH "People of Color")) OR (ethnic* or racial* or race or cultur* or minority* or immigrant* or migrant* or native or Indigenous or Aboriginal or “First Nation*” or “African American” or “American Indian” or “Mexican American” or Black or White or Caucasian or European or Asian or Maori or Hispanic or Latino or Caribbean or “Middle Eastern” or Chinese).ti,ab.) AND (Publication date: 20,140,101–20241231)	02/12/2024
Scopus	TITLE-ABS (diabet* OR prediabet* OR "impaired glucose tolerance" OR "impaired fasting glucose" OR t1d* OR t2d*) AND TITLE-ABS (adult* OR "middle-aged" OR aged OR person* OR people OR patient*) AND TITLE-ABS ("mental illness" OR "mental health disorder" OR "mental problem" OR "mental disorder" OR "mental disease" OR psychiatric OR "psychotic disorder" OR psychosis OR psychotrauma OR "mood disorder" OR schizophrenia OR "delusional disorder" OR depressi* OR "bipolar disorder" OR anxiety OR "panic disorder" OR "obsessive–compulsive disorder" OR "post-traumatic stress disorder" OR "eating disorder" OR "anorexia nervosa" OR "bulimia nervosa" OR "binge-eating disorder" OR "personality disorder") AND TITLE-ABS (ethnic* OR racial* OR race OR cultur* OR minority* OR immigrant* OR migrant* OR native OR indigenous OR aboriginal OR "First Nation*" OR "African American" OR "American Indian" OR "Mexican American" OR black OR white OR caucasian OR european OR asian OR maori OR hispanic OR latino OR caribbean OR "Middle Eastern" OR chinese) AND PUBYEAR > 2013 AND PUBYEAR < 2024	17/12/2024

### Eligibility Criteria

This review included studies published between January 2014 and December 2024 that reported the prevalence of any mental health disorder among adults (aged 18 years or older) with diabetes across ethnic groups. The time frame for the reviewed studies was selected to align with the publication of updated DSM-5 guidelines [23] and contemporary shifts in diabetes classification and management. The selected time frame also captures studies using modern, consistent diagnostic frameworks for mental health disorders. Only peer-reviewed studies published in English were considered to ensure quality, accessibility, and consistency. Studies were excluded if they focused on pregnant women, gestational diabetes, or individuals with conditions such as autism spectrum disorder or intellectual disabilities, as these populations differ in clinical and psychological profiles and could confound prevalence estimates. Non-peer-reviewed literature (e.g., editorials, letters, case reports, grey literature) and studies not primarily designed to report prevalence, such as clinical trials, and protocols, were also excluded to maintain methodological consistency and relevance to the review objective.

### Study Selection

**Fig. 1:**
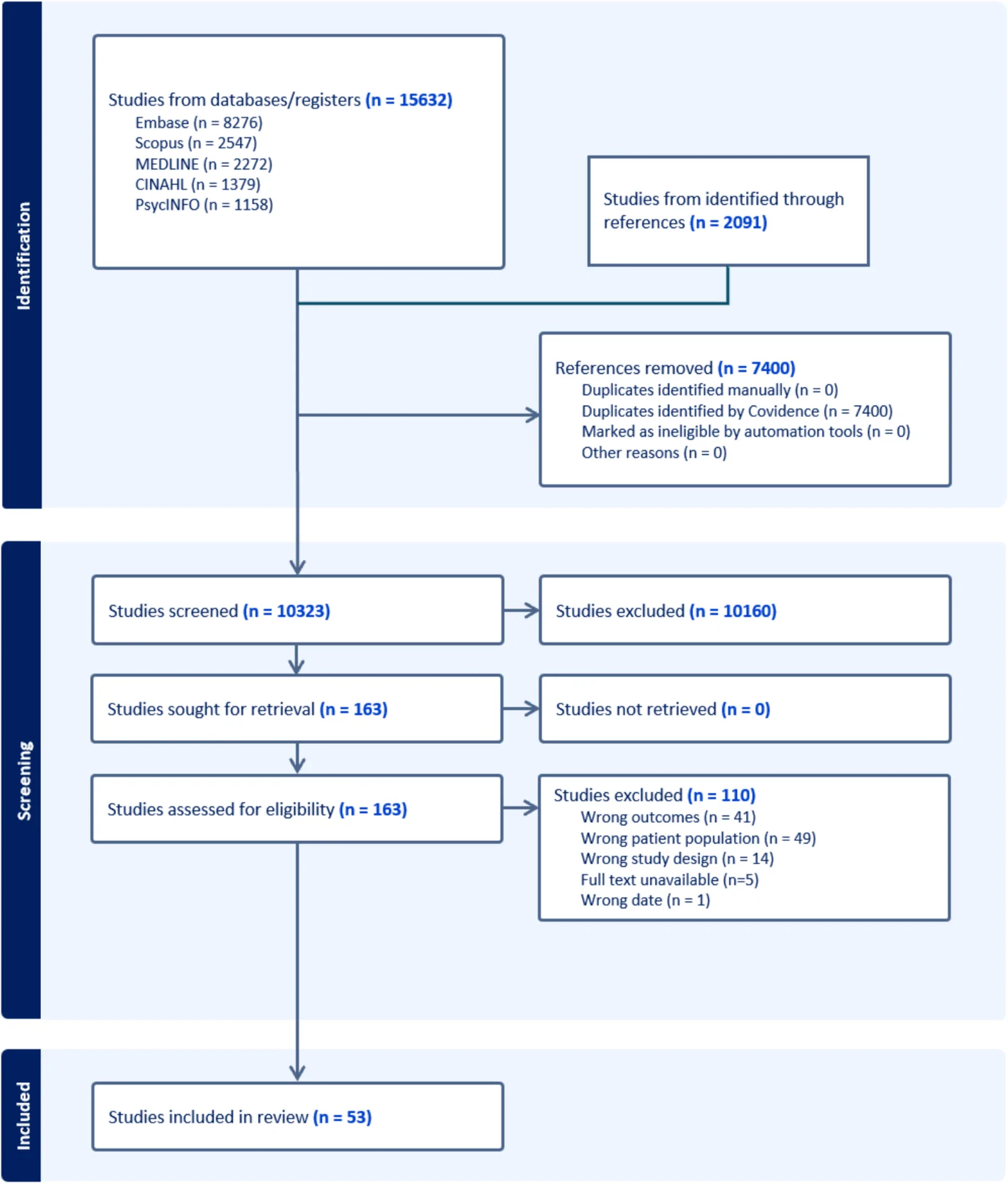
PRISMA Flowchart of the study selection process

Two independent reviewers (ML and SJ) screened titles and abstracts for eligibility, followed by full-text assessments. Disagreements were resolved through consensus or by a third reviewer NEE. The process was conducted via Covidence, with results summarized in a PRISMA flow diagram (Fig. 1).

### Data Extraction

ML, SJ and NEE did the data extraction and was then independently verified by two authors, KA and ULO. A standardized data extraction format was employed in Covidence, which included the following information: lead author’s surname, year of publication, sample size, age or age group, ethnicity (considering immigration/migrant status, Indigenous status, and country of origin), type of diabetes, duration of diabetes, type of mental health disorders assessed, method used in diagnosing mental health disorders, diagnostic and prevalence rates of mental health disorders stratified by ethnicity. Ethnicity was categorized according to the definitions used in the reviewed studies and aligned with the World Bank’s regional classifications. The ethnic groups included Indigenous (Torres Strait Islanders, Aboriginals, First Nations), Caucasians (Europeans, White Australia-born, and North Americans), Asians (Indian, Bangladeshi, Chinese, Japanese, Filipino, etc.), Middle Eastern and North African (Egyptians, Syrians, Turkish), South and Central American or Latinos/Hispanics (Mexicans, Brazilians, Chileans, Peruvians etc.), Africa origins (Sub-Sahara Africans, Black or African Americans, Afro Caribbeans), Oceanian (Samoa, Tuvalu, Vanuatu) and Mixed/Brown or unspecified (combination of Black, Asian and White groups). In cases where studies reported multiple ethnicities without specific data for each group, the predominant ethnicity (representing at least 80% of the participants) was used for classification. Discrepancies between reviewers were resolved by a third author (AKD). Studies were selected for analysis after confirming they met the inclusion criteria from online sources. Following data extraction, the Excel sheet from Covidence was downloaded and cleaned for data analysis.

### Quality Assessment

Three authors (ML, SJ, and NE) independently examined the included studies using the AXIS tool for cross-sectional studies [24], and Newcastle–Ottawa Scale (NOS) for cohort studies [25, 26]. The tools are attached as appendix 2 and 3. The AXIS tool, comprising 20 items across 5 domains (introduction, methods, results, discussion, and other), was used to assess the overall rating of each study as a rating out of 100. Reviewers responded ‘Y’ if the criteria were adequately met, ‘N’ if the criteria were not adequately met, and ‘U’ if they were unsure about the criteria. A percentage score was derived by calculating the number of ‘Yes’ responses out of the total applicable items. Based on these scores, studies were classified as high quality (‘Good’, ≥ 70%), moderate quality (‘Fair’, 50–69%), or low quality (‘Poor’, < 50%). Studies were considered low risk if they met ≥ 50% of quality indicators. Disagreements were resolved with input from two additional reviewers (AKD and KA).

### Risk of Bias Assessment

Three authors (ML, SJ, and NE) independently assessed the risk of bias using a validated bias assessment tool [27], consisting of ten items that evaluate four domains of bias, internal and external validity. Disagreements raised during the assessment were resolved by discussion with the third reviewer (ULO). The first four items (items 1–4) evaluate the presence of selection bias, non-response bias, and external validity. The remaining six items (items 5–10) assess the presence of biases in measurement and analysis, and internal validity. Studies were rated as low risk: ≥ 8 “Yes” responses, moderate risk: 6–7 “Yes” responses, and high risk: ≥ 5 Yes” responses as reported in the supplementary file (Supplementary Material 3).

### Data Analysis

Data were analyzed using Stata version 17. The ‘metaprop’ command was used to calculate pooled prevalence estimates with 95% confidence intervals, stratified by ethnicity. Heterogeneity was assessed using Cochran’s Q test and the I^2^ statistic, with thresholds categorised as high (I^2^ ≥ 75%), moderate (I^2^ ≥ 50%), or low (I^2^ ≥ 25%). The ‘metan’ command was used to calculate the pooled mean age of individuals with diabetes and mental health disorders (see Fig. 9). Publication bias was evaluated using funnel plots and Egger’s test.

### Subgroup and Meta-Regression Analyses

Subgroup analyses were performed by ethnicity, mean age, type of diabetes, type of mental health disorder, diagnostic method, duration of diabetes, Indigenous status, region, year of publication, and country income level. Mental health diagnostic methods were categorized as questionnaire-based or clinical/non-questionnaire-based [28]. Ethnicity was grouped into the following categories: Asian, African, Middle East and North Africa, European descent/White, Pacific, Hispanic, Mixed/Brown (combination of Black, Asian and White groups) [29] and Indigenous populations [30, 31]. Indigenous status was assigned based on studies in countries that have indigenous people such as the USA, Canada, New Zealand, and Australia [32]. Country income levels were determined using the World Bank’s classification of national income groups [33, 34]. Duration of Diabetes was categorized as either less than 10 years or 10 years and above. This threshold was used because the 10-year mark often reflects a critical point at which the risk of developing chronic complications and mental health disorders increases significantly. Additionally, using this binary classification helped to simplify analysis and allows for clearer comparisons between individuals with shorter versus longer disease exposure.

Mental health disorder diagnosis was categorized based on two approaches: (1) standardized self-report questionnaires or screening tools (e.g., PHQ-9, GAD-7), which identified probable cases using established cutoff scores, and (2) clinical assessments, medical records, or diagnostic interviews conducted by qualified professionals, which indicated confirmed diagnoses. The classification of mental health disorders was based on the most commonly studied disorders as identified in previous literature [35–37]. Diabetes was classified as type 1, type 2, both types, and unspecified.

Meta-regression using the **‘**metareg’ command** evaluated interactions between key variables, including type of mental health disorder, method of diagnosis, indigenous status, ethnicity, type of diabetes, level of income of country, duration of diabetes, age, region and year of publication. A leave-one-out sensitivity analysis was conducted to assess the robustness of pooled prevalence estimates by examining the effect of omitting a single study on the overall prevalence.

## Results

### Study Selection

The database search yielded 15,632 results, and citation tracking produced a total of 2,091 records, of which 7,400 were duplicates and so were not included in the review. After removing the duplicates, titles, and abstracts of 10,323 studies were screened. A total of 10,160 studies were excluded because they did not meet the eligibility criteria. Of the remaining 163 studies that underwent full-text screening, only 53 were included in the systematic review and meta-analysis. The details of the study selection process are presented in the PRISMA flowchart (Fig. 1).

### Systematic Review of Selected Studies

#### Description of Study Characteristics

Table 2 presents the characteristics of the studies included in the final review. Most of the studies were cross-sectional (*n* = 42), while the remainder were cohort studies (*n* = 11). The studies were predominantly conducted in the United States (*n* = 35), followed by Hong Kong (*n* = 3), Australia (*n* = 3), and England (*n* = 3). The populations examined were mainly Africans or Black Americans (*n* = 24), Caucasians or White (*n* = 21), Hispanic (*n* = 11), and Asian (*n* = 3). Most studies focused on patients diagnosed exclusively with Type 2 Diabetes Mellitus (T2DM) (*n* = 30), followed by studies involving both Type 1 and Type 2 diabetes (*n* = 6), and those focusing exclusively on Type 1 diabetes mellitus (T1DM) (*n* = 3). The remaining 14 studies did not specify the type of diabetes under investigation. The studies included a broad range of sample sizes, from 41 to 2,722,450 participants, with a mean age spanning from 37 to 72 years. The duration of diabetes in the sample populations ranged from 5.3 to 21 years. A large proportion of the studies (*n* = 50) examined the prevalence of depression or depressive symptoms, with the most used assessment tools being the PHQ-9 (Patient Health Questionnaire) (*n* = 13), CES-D (Center for Epidemiological Studies Depression Scale) (*n* = 11), and PHQ-8 (*n* = 4). Other studies focused on other mental health conditions, including anxiety (*n* = 5), post-traumatic stress disorder (PTSD) (*n* = 4), schizophrenia (*n* = 1), and various other mental health disorders (unclassified) (*n* = 3).

**Table 2:** Characteristics of included studies

First author	Year	Country	Study design	Type of mental health disorders assessed	Sample size (n)	Ethnicity studied	Prevalence of the mental health disorder (n, %)	Mean age (n, SD)	Mental health diagnostic assessment tool used	Duration of diabetes (mean, SD)	Type of diabetes included	Quality assessment
Musselman et al. [38]	2014	USA	Cross-sectional	Major depressive disorder	172	African American	33, 19%	50	ZDRS	2.9 (median)	Type 2	65
Musselman et al. [38]	2014	USA	Cross-sectional	Anxiety disorder (panic disorder, agoraphobia, social phobia, PTSD, OCD, GAD)	172	African American	24, 14%	50	MINI, TEI	2.9 (median)	Type 2	65
Vanderlip et al. [39]	2014	USA	Cross-sectional	Depression	133	White	32, 24.06% (*p* = 0.071)	57.03	PHQ-9, Anxiety and Depression Detector, GAD-7, AUDIT-C		Unspecified	80
Lee et al. [40]	2020	USA	Cross-sectional	Depression	4,233	White	PHQ-8 ≥ 10: 566, 72.3% (*p* = 0.464) PHQ-8 ≥ 5: 1296, 71.2% (*p* = 0.321)		PHQ-8		Type 1 or 2	70
Lee et al. [40]	2020	USA	Cross-sectional	Depression	505	Hispanic	PHQ-8 ≥ 10: 77, 9.8% (*p* = 0.464) PHQ-8 ≥ 5: 177, 9.7% (*p* = 0.321)		PHQ-8		Type 1 or 2	70
Lee et al. [40]	2020	USA	Cross-sectional	Depression	778	Black	PHQ-8 ≥ 10: 97, 12.4% (*p* = 0.464) PHQ-8 ≥ 5: 240, 13.2% (*p* = 0.321)		PHQ-8		Type 1 or 2	70
Dibato et al. [41]	2023	USA	Cohort	Depression	2,722,450 (920,771 T2DM, 1,801,679 T1DM)	African American	24,434, 17%	56	DSM-5		Type 2	Good
Dibato et al. [41]	2023	USA	Cohort	Depression	2,722,450 (920,771 T2DM, 1,801,679 T1DM)	White Caucasian	219,070, 28%	60	DSM-5		Type 2	Good
Dibato et al. [41]	2023	USA	Cohort	Mental illness (depression, anxiety, bipolar, schizophrenia, PTSD, eating, gender identity, personality, hysteria, others)	2,722,450 (920,771 T2DM, 1,801,679 T1DM)	African American	35,758, 26%	56	DSM-5		Type 2	Good
Dibato et al. [41]	2023	USA	Cohort	Mental illness (depression, anxiety, bipolar, schizophrenia, PTSD, eating, gender identity, personality, hysteria, others)	2,722,450 (920,771 T2DM, 1,801,679 T1DM)	White Caucasian	285,277, 37%	60	DSM-5		Type 2	Good
Munoz et al. [42]	2014	Mexico	Cross-sectional	Depression	241	Mexican	CES-D ≥ 16: 76, 31.5% (*p* = 0.0005) CES-D ≥ 23: 42, 17.4% (*p* < 0.0001)	52.5, 5.67 (*p* = 0.4182)	CES-D	10.13, 7.27	Type 2	70
Munoz et al. [42]	2014	USA	Cross-sectional	Depression	92	Mexican American	CES-D ≥ 16: 48, 52% (*p* = 0.0005) CES-D ≥ 23: 35, 38% (*p* < 0.0001)	51.6, 7.58	CES-D	9.75, 8.06	Type 2	70
Huyser et al. [43]	2020	USA	Cross-sectional	Depression	437,608	American Indian & Alaska Native	6,815, 15.66%		ICD-9-CM		Unspecified	60
Huyser et al. [43]	2020	USA	Cross-sectional	Mental health disorder (depression, anxiety, bipolar, PTSD)	437,608	American Indian & Alaska Native	10,214, 23.47%		ICD-9-CM		Unspecified	60
Ismail et al. [44]	2017	England	Cohort	Depressive symptoms	840	White	116, 13.81% (*p* = 0.77)	56.2, 11.1 (nondepressed: 56.72, 11.07, depressed: 53.07, 10.47)	PHQ-9 (≥ 10)		Type 2	Good
Ismail et al. [44]	2017	England	Cohort	Depressive symptoms	643	Black	82, 12.75% (*p* = 0.77)	56.2, 11.1 (nondepressed: 56.72, 11.07, depressed: 53.07, 10.47)	PHQ-9 (≥ 10)		Type 2	Good
Ismail et al. [44]	2017	England	Cohort	Depressive symptoms	168	South Asian/other	34, 20.24% (*p* = 0.77)	56.2, 11.1 (nondepressed: 56.72, 11.07, depressed: 53.07, 10.47)	PHQ-9 (≥ 10)		Type 2	Good
Meng et al. [45]	2014	Australia	Cross-sectional	Depression	100	Chinese Australian	PHQ-9 ≥ 5: mild to severe 42, 42% PHQ-9 > 10: mild to severe 19, 19%	Minimal: 61.4, 13.4 Mild: 63.5, 12.3 Moderate to severe: 62.5, 16.7	PHQ-9	< 10: 34, 34%, ≥ 10: 66, 66%	Type 1 or 2	75
Brown et al. [46]	2017	USA	Cross-sectional	Depression/anxiety	2,255	Caucasian	367, 16.27% (*p* = 0.143)	62.2, 13.5 (with MHC: 58.6, 13.6, without MHC: 63.3, 13.2)	ICD-9		Type 2	65
Brown et al. [46]	2017	USA	Cross-sectional	Serious mental illness (bipolar, psychosis, schizophrenia, psychotic)	2,255	Caucasian	202, 8.96%	62.2, 13.5 (with MHC: 58.6, 13.6, without MHC: 63.3, 13.2)	ICD-9		Type 2	65
Brown et al. [46]	2017	USA	Cross-sectional	Depression/anxiety	62	African American	7, 11.29% (*p* = 0.143)	62.2, 13.5 (with MHC: 58.6, 13.6, without MHC: 63.3, 13.2)	ICD-9		Type 2	65
Brown et al. [46]	2017	USA	Cross-sectional	Serious mental illness (bipolar, psychosis, schizophrenia, psychotic)	62	African American	11, 17.74%	62.2, 13.5 (with MHC: 58.6, 13.6, without MHC: 63.3, 13.2)	ICD-9		Type 2	65
Tung et al. [47]	2015	Hong Kong	Cross-sectional	Depressive (MDD, dysthymic, adjustment)	136	Hong Kong Chinese	32, 23.5%	39.8, 12.1	HADS	15.6, 10.4	Type 1	75
Tung et al. [47]	2015	Hong Kong	Cross-sectional	Anxiety (panic, agoraphobia, social phobia, OCD, PTSD, GAD, adjustment)	136	Hong Kong Chinese	35, 25.7%	39.8, 12.1	HADS	15.6, 10.4	Type 1	75
Tung et al. [47]	2015	Hong Kong	Cross-sectional	Schizophrenia	136	Hong Kong Chinese	2, 1.5%	39.8, 12.1	HADS	15.6, 10.4	Type 1	75
Trief et al. [48]	2014	USA	Cross-sectional	Depression	5,491	White non-Hispanic	PHQ-8 ≥ 10: 465, 8.47% (*p* < 0.001) PHQ-8 ≥ 12: 349, 6.36% (*p* < 0.001)	34 (median)	PHQ-8	16 (median)	Type 1	75
Trief et al. [48]	2014	USA	Cross-sectional	Depression	174	Black non-Hispanic	PHQ-8 ≥ 10: 22, 12.64% (*p* < 0.001) PHQ-8 ≥ 12: 16, 9.20% (*p* < 0.001)	34 (median)	PHQ-8	16 (median)	Type 1	75
Trief et al. [48]	2014	USA	Cross-sectional	Depression	316	Hispanic or Latino	PHQ-8 ≥ 10: 57, 18.04% (*p* < 0.001) PHQ-8 ≥ 12: 40, 12.66% (*p* < 0.001)	34 (median)	PHQ-8	16 (median)	Type 1	75
Andrade et al. [49]	2023	Brazil	Cross-sectional	Depressive symptoms	151	Brown	34, 22.52% (*p* = 0.037)	71.6, 8.03	GDS-15		Unspecified	70
Andrade et al. [49]	2023	Brazil	Cross-sectional	Depressive symptoms	43	Black	9, 20.93% (*p* = 0.037)	71.6, 8.03	GDS-15		Unspecified	70
Andrade et al. [49]	2023	Brazil	Cross-sectional	Depressive symptoms	39	White	11, 28.21% (*p* = 0.037)	71.6, 8.03	GDS-15		Unspecified	70
Laake et al. [50]	2014	England	Cross-sectional	Depression	878	White	128, 14.58% (*p* = 0.128)	56.1, 11.04	PHQ-9 (≥ 10)		Type 2	65
Laake et al. [50]	2014	England	Cross-sectional	Depression	710	Black	95, 13.38% (*p* = 0.128)	56.1, 11.04	PHQ-9 (≥ 10)		Type 2	65
Laake et al. [50]	2014	England	Cross-sectional	Depression	181	Asian/other	35, 19.34% (*p* = 0.128)	56.1, 11.04	PHQ-9 (≥ 10)		Type 2	65
Zhang et al. [51]	2015	Hong Kong	Cross-sectional	Depression	545	Hong Kong Chinese	CES-D ≥ 21 97, 17.8% PHQ-9 ≥ 7 96, 17.6%	54.6, 9.5	CES-D, PHQ-9	6	Type 2	70
Wang et al. [52]	2016	USA	Cross-sectional	Depression	1,318	White non-Hispanic	CRD: 140, 10.62% CSD: 130, 9.86%		PHQ-9 (≥ 10)	9.9, 0.2	Type 2	80
Wang et al. [52]	2016	USA	Cross-sectional	Depression	388	Black non-Hispanic	CRD: 55, 14.18% CSD:60, 15.46%		PHQ-9 (≥ 10)	9.9, 0.2	Type 2	80
Wang et al. [52]	2016	USA	Cross-sectional	Depression	297	Mexican & other Hispanic	CRD: 51, 17.17% CSD:56, 18.86%		PHQ-9 (≥ 10)	9.9, 0.2	Type 2	80
Sorkin et al. [53]	2015	USA	Cross-sectional	Depressive symptoms	120	White non-Hispanic	63, 52.5% (*p* = 0.67)	Low depressive symptoms: 58.0, 11.2 High depressive symptoms: 57.7, 11.2	CES-D		Type 2	85
Sorkin et al. [53]	2015	USA	Cross-sectional	Depressive symptoms	439	Hispanic	230, 52.39% (*p* = 0.21)	Low depressive symptoms: 58.0, 11.2 High depressive symptoms: 57.7, 11.2	CES-D		Type 2	85
Sorkin et al. [53]	2015	USA	Cross-sectional	Depressive symptoms	151	Vietnamese	92, 60.93% (*p* = 0.06)	Low depressive symptoms: 58.0, 11.2 High depressive symptoms: 57.7, 11.2	CES-D		Type 2	85
Langberg et al. [54]	2022	USA	Cross-sectional	Depression	180	White non-Hispanic	119, 76.1%		PHQ-9 (≥ 5)		Unspecified	60
Langberg et al. [54]	2022	USA	Cross-sectional	Depression	88	Black non-Hispanic	67, 76.1%		PHQ-9 (≥ 5)		Unspecified	60
Langberg et al. [54]	2022	USA	Cross-sectional	Depression	71	Hispanic	57, 84.1%		PHQ-9 (≥ 5)		Unspecified	60
Trief et al. [55]	2019	USA	Cohort	Depressive symptoms	2,518	White non-Hispanic	PHQ-8 ≥ 10: 222, 8.8% BRFSS: 107, 4.2%	42, 16	PHQ-8 (≥ 10), BRFSS	21	Type 1	Good
Trief et al. [55]	2019	USA	Cohort	Depressive symptoms	62	Black non-Hispanic	PHQ-8 ≥ 10: 8, 12.9% BRFSS:4, 6.5%	42, 16	PHQ-8 (≥ 10), BRFSS	21	Type 1	Good
Trief et al. [55]	2019	USA	Cohort	Depressive symptoms	88	Hispanic or Latino	PHQ-8 ≥ 10: 15, 17.0% BRFSS:8, 9.1%	42, 16	PHQ-8 (≥ 10), BRFSS	21	Type 1	Good
Dibato et al. [56]	2021	USA	Cohort	Depression	505,336	White Caucasian	157,116, 31.09%		DSM-5		Type 2	Good
Dibato et al. [56]	2021	USA	Cohort	Depression	91,104	African American	19,300, 21.18%		DSM-5		Type 2	Good
Breland et al. [57]	2023	USA	Cohort	Depression	8,169	American Indian or Alaska Native	25%		FY17, VHA’s WHEI		Type 1 or 2	60
Breland et al. [57]	2023	USA	Cohort	Depression	7,740	Asian	19%		FY17, VHA’s WHEI		Type 1 or 2	60
Breland et al. [57]	2023	USA	Cohort	Depression	176,765	Black or African American	24%		FY17, VHA’s WHEI		Type 1 or 2	60
Breland et al. [57]	2023	USA	Cohort	Depression	57,766	Hispanic or Latino/Latina	26%		FY17, VHA’s WHEI		Type 1 or 2	60
Breland et al. [57]	2023	USA	Cohort	Depression	7,115	Native Hawaiian or Other Pacific Islander	24%		FY17, VHA’s WHEI		Type 1 or 2	60
Breland et al. [57]	2023	USA	Cohort	Depression	546,450	White	22%		FY17, VHA’s WHEI		Type 1 or 2	60
Dixon et al. [58]	2019	USA	Cross-sectional	PTSD	290	African American	80, 30.4%	51.5, 9.6	MPSS		Type 2	65
Dixon et al. [58]	2019	USA	Cross-sectional	Depression	290	African American	76, 29.3%	51.5, 9.6	BDI		Type 2	65
Fung et al. [59]	2018	Hong Kong	Cross-sectional	Depressive symptoms	325	Hong Kong Chinese	42, 13%	69 (median)	GDS	12.0, 8.3	Type 2	70
Wang et al. [60]	2021	China	Cross-sectional	Depressive symptoms	1,288	Han	307, 23.84%	66.9	CES-D (≥ 16)	8.4, 7.6	Type 2	70
Taylor et al. [61]	2017	Australia	Cross-sectional	Depression	188	Torres Strait Islander	Mild: 86, 45.7%, Moderate-severe: 23, 12.2%	58.2	PHQ-9	9.5	Type 2	65
Seaton et al. [62]	2019	USA	Cross-sectional	Depression	96	Marshallese	10, 10.42%	54, 11.0	PHQ-2 (≥ 3)	5.3, 8.5	Type 1 or 2	50
Morales et al. [63]	2023	USA	Cross-sectional	Depression	213	Mexican American	57, 26.8%		Self-reported		Type 1 or 2	70
Morales et al. [63]	2023	USA	Cross-sectional	Anxiety	213	Mexican American	58, 27.2%		Self-reported		Type 1 or 2	70
Wu et al. [64]	2023	China	Cohort	Depression	2933	Ethnic minority	164, 5.95% (*p* < 0.001)	43.63, 14.84	PHQ-9 (≥ 5)		Type 2	Good
Joshi et al. [65]	2015	Nepal	Cross-sectional	Depressive symptoms	61	Chhetri	24, 39.34% (*p* = 0.005)	54.8, 10.6	BDI-II (≥ 14)	8.7 (median)	Type 2	80
Joshi et al. [65]	2015	Nepal	Cross-sectional	Depressive symptoms	87	Brahman	40, 45.98% (*p* = 0.005)	54.8, 10.6	BDI-II (≥ 14)	8.7 (median)	Type 2	80
Joshi et al. [65]	2015	Nepal	Cross-sectional	Depressive symptoms	178	Newar	76, 42.70% (*p* = 0.005)	54.8, 10.6	BDI-II (≥ 14)	8.7 (median)	Type 2	80
Joshi et al. [65]	2015	Nepal	Cross-sectional	Depressive symptoms	38	Janajati	22, 57.89% (*p* = 0.005)	54.8, 10.6	BDI-II (≥ 14)	8.7 (median)	Type 2	80
Laseter et al. [66]	2013	USA	Cohort	Depression	16,760	Black	90% (*p* < 0.0001)	66.5, 9.90	ICD-9		Type 2	Good
Laseter et al. [66]	2013	USA	Cohort	Depression	83,591	White	91% (*p* < 0.0001)	66.5, 9.90	ICD-9		Type 2	Good
Choi et al. [67]	2013	USA	Cross-sectional	Depressive symptoms	164	Korean American	56.10%	68.18, 9.30	CES-D (≥ 16)	10.80, 9.86	Type 2	70
Kimbro et al. [68]	2014	USA	Cross-sectional	Depression	1890	White	329, 17.41% (*p* < 0.01)		PHQ-8 (≥ 9)	Nondepressed: 13.0 Depressed: 13.3	Unspecified	70
Kimbro et al. [68]	2014	USA	Cross-sectional	Depression	532	Hispanic	95, 17.86% (*p* < 0.01)		PHQ-8 (≥ 9)	Nondepressed: 13.0 Depressed: 13.3	Unspecified	70
Kimbro et al. [68]	2014	USA	Cross-sectional	Depression	599	Black	135, 22.54% (*p* < 0.01)		PHQ-8 (≥ 9)	Nondepressed: 13.0 Depressed: 13.3	Unspecified	70
Kimbro et al. [68]	2014	USA	Cross-sectional	Depression	131	Asian/Pacific Islander	12, 9.16% (*p* < 0.01)		PHQ-8 (≥ 9)	Nondepressed: 13.0 Depressed: 13.3	Unspecified	70
Karter et al. [69]	2015	USA	Cohort	Depression	11,417	African American	1,450, 12.70% (*p* < 0.05)	72	ICD-9		Type 1 or 2	Good
Karter et al. [69]	2015	USA	Cohort	Depression	11,275	Asian	960, 8.51% (*p* < 0.05)	72	ICD-9		Type 1 or 2	Good
Karter et al. [69]	2015	USA	Cohort	Depression	8,162	Filipino	649, 7.95% (*p* < 0.05)	72	ICD-9		Type 1 or 2	Good
Karter et al. [69]	2015	USA	Cohort	Depression	14,234	Latino	2,550, 17.91% (*p* < 0.05)	72	ICD-9		Type 1 or 2	Good
Karter et al. [69]	2015	USA	Cohort	Depression	63,509	White non-Hispanic	12,381, 19.49% (*p* < 0.05)	72	ICD-9		Type 1 or 2	Good
Karter et al. [69]	2015	USA	Cohort	Depression	6,851	Mixed race	1,445, 21.09% (*p* < 0.05)	72	ICD-9		Type 1 or 2	Good
Aronson et al. [70]	2016	USA	Cross-sectional	PTSD	206	American Indian	45, 21.8% (*p* < 0.0001)	56.5	PC-PTSD (≥ 3)	14.7	Type 2	70
Aronson et al. [70]	2016	USA	Cross-sectional	Depression	206	American Indian	35, 17.1% (*p* < 0.0001)	56.5	PHQ-9 (≥ 10)	14.7	Type 2	70
Kalyani et al. [71]	2017	USA	Cross-sectional	Depressive symptoms	631	African American	164, 25.99% (*p* < 0.0001)	54.5, 12.5	CES-D (≥ 16)		Unspecified	80
Olvera et al. [72]	2016	USA	Cross-sectional	Depression	335	Mexican American	123, 41.3% (*p* < 0.0001)	58	CES-D (≥ 16)		Unspecified	75
O'Connell et al. [73]	2021	USA	Cross-sectional	Depression	43,518	American Indian & Alaska Native	6,815, 15.66%		ICD-9		Unspecified	55
O'Connell et al. [73]	2021	USA	Cross-sectional	Other mental health disorders (anxiety, bipolar, PTSD)	43,518	American Indian & Alaska Native	5,752, 13.22%		ICD-9		Unspecified	55
Davis et al. [74]	2015	Australia	Cross-sectional	Depression	52	Aboriginal	Any current: 13, 25.0% (*p* = 0.16) Major current: 8, 15.4% (*p* = 0.029) Lifetime: 19, 41.3% (*p* = 0.19)	54.3, 11.8	PHQ-9 (≥ 5), BLDS	8	Type 2	80
Davis et al. [74]	2015	Australia	Cross-sectional	Depression	772	Anglo-Celt	Any current: 82 (*p* = 0.16), 10.6% Major current: 32, 4.1% (*p* = 0.029) Lifetime: 262, 34.7% (*p* = 0.19)	67.2, 10.6	PHQ-9 (≥ 5), BLDS	8	Type 2	80
Sidhu et al. [75]	2016	Canada	Cross-sectional	Depression	41	South Asian Canadian	18, 43.90% (*p* = 0.04)	67, 9	PHQ-9 (≥ 10)		Type 2	50
Chew et al. [76]	2016	Malaysia	Cross-sectional	Depression	361	Malay	Mild: 95, 26.32 Moderate-severe: 45, 12.47%	56.9	PHQ-9	4.0 (median)	Type 2	65
Chew et al. [76]	2016	Malaysia	Cross-sectional	Depression	157	Chinese	Mild: 49, 31.21% Moderate-severe: 19, 12.10%	56.9	PHQ-9	4.0 (median)	Type 2	65
Chew et al. [76]	2016	Malaysia	Cross-sectional	Depression	161	Indian	Mild: 61, 37.89% Moderate-severe: 12, 7.45%	56.9	PHQ-9	4.0 (median)	Type 2	65
Kuo et al. [77]	2020	USA	Cross-sectional	Depressive symptoms	72	Mexican American	23, 31.9% (*p* = 0.514)	53.85, 13.44	CES-D (≥ 16)	8.59, 7.47	Type 2	60
Kuo et al. [77]	2020	USA	Cross-sectional	Depressive symptoms	134	Chinese American	37, 27.6% (*p* = 0.514)	69.01, 10.66	CES-D (≥ 16)	13.18, 9.97	Type 2	60
Huang et al. [78]	2021	USA	Cross-sectional	Depressive symptoms	151	Chinese American	28.5% (*p* < 0.001)	69.91, 9.50	CES-D (≥ 16)	13.66, 10.21	Type 2	70
Cummings et al. [79]	2022	USA	Cohort	Depressive symptoms	1,560	Black	226, 14.49%	Nondepressed: 64.5, 8.3, Depressed: 62.6, 8.5	CES-D (≥ 4)		Unspecified	Good
Scherrer et al. [80]	2024	USA	Cohort	PTSD	3162	Black	2594, 82.04%		ICD-9 or ICD-10		Type 2	Good
Scherrer et al. [80]	2024	USA	Cohort	PTSD	6267	White	4812, 76.79%		ICD-9 or ICD-10		Type 2	Good
Scherrer et al. [80]	2024	USA	Cohort	PTSD	573	Other (American Indian or Alaska Native, Asian or Native Hawaiian or Other Pacific Islander)	472, 82.37%		ICD-9 or ICD-10		Type 2	Good
Valentine et al. [81]	2017	USA	Cross-sectional	PTSD	199	Latino	28, 14.07%		WHM-CIDI		Unspecified	65
Valentine et al. [81]	2017	USA	Cross-sectional	PTSD	112	US-born Latino	13, 11.61%	37	WHM-CIDI		Unspecified	65
Valentine et al. [81]	2017	USA	Cross-sectional	PTSD	87	Foreign-born Latino	15, 17.24%	38.9	WHM-CIDI		Unspecified	65
Valentine et al. [81]	2017	USA	Cross-sectional	PTSD	429	Non-Latino White	25, 5.83%	46.9	WHM-CIDI		Unspecified	65
Johnson et al. [82]	2020	USA	Cross-sectional	Depression	212	Mexican American	85, 40.09%	60	GDS		Unspecified	65
Kendzor et al. [83]	2015	USA	Cross-sectional	Depression	486	Mexican American	141, 29.01%	51.3, 14.6	CES-D (≥ 16)		Unspecified	75
Kendzor et al. [83]	2015	USA	Cross-sectional	Anxiety	486	Mexican American	124, 25.51%	51.3, 14.6	ZSAS (≥ 45)		Unspecified	75
Munshi et al. [84]	2021	England	Cohort	Severe mental illness (schizophrenia-spectrum, bipolar affective, manic episode, severe depressive episode with psychotic symptoms)	12,941	White British	578, 4.47% (*p* < 0.001)	T2DM only: 63, 14 Comorbid: 60, 13	ICD-10	8.7	Type 2	Good
Munshi et al. [84]	2021	England	Cohort	Severe mental illness (schizophrenia-spectrum, bipolar affective, manic episode, severe depressive episode with psychotic symptoms)	423	Irish	24, 5.67% (*p* < 0.001)	T2DM only: 63, 14 Comorbid: 60, 13	ICD-10	8.7	Type 2	Good
Munshi et al. [84]	2021	England	Cohort	Severe mental illness (schizophrenia-spectrum, bipolar affective, manic episode, severe depressive episode with psychotic symptoms)	7,766	Black African	326, 4.20% (*p* < 0.001)	T2DM only: 63, 14 Comorbid: 60, 13	ICD-10	8.7	Type 2	Good
Munshi et al. [84]	2021	England	Cohort	Severe mental illness (schizophrenia-spectrum, bipolar affective, manic episode, severe depressive episode with psychotic symptoms)	7,165	Black Caribbean	455, 6.35% (*p* < 0.001)	T2DM only: 63, 14 Comorbid: 60, 13	ICD-10	8.7	Type 2	Good
Munshi et al. [84]	2021	England	Cohort	Severe mental illness (schizophrenia-spectrum, bipolar affective, manic episode, severe depressive episode with psychotic symptoms)	16,674	Bangladeshi	569, 3.41% (*p* < 0.001)	T2DM only: 63, 14 Comorbid: 60, 13	ICD-10	8.7	Type 2	Good
Munshi et al. [84]	2021	England	Cohort	Severe mental illness (schizophrenia-spectrum, bipolar affective, manic episode, severe depressive episode with psychotic symptoms)	6,886	Indian	189, 2.74% (*p* < 0.001)	T2DM only: 63, 14 Comorbid: 60, 13	ICD-10	8.7	Type 2	Good
Munshi et al. [84]	2021	England	Cohort	Severe mental illness (schizophrenia-spectrum, bipolar affective, manic episode, severe depressive episode with psychotic symptoms)	4,429	Pakistani	110, 2.48% (*p* < 0.001)	T2DM only: 63, 14 Comorbid: 60, 13	ICD-10	8.7	Type 2	Good
Munshi et al. [84]	2021	England	Cohort	Severe mental illness (schizophrenia-spectrum, bipolar affective, manic episode, severe depressive episode with psychotic symptoms)	486	Chinese	2, 4.32% (*p* < 0.001)	T2DM only: 63, 14 Comorbid: 60, 13	ICD-10	8.7	Type 2	Good
Briganti et al. [85]	2018	Brazil	Cross-sectional	Depressive symptoms	1,556	White	Mild: 12.98% Moderate: 4.47% Moderately severe: 2.08% Severe: 0.94%		PHQ-9	0–2: 27.8% 3–7: 24.8% 8–15: 23.5% ≥ 16: 23.9%	Unspecified	75
Briganti et al. [85]	2018	Brazil	Cross-sectional	Depressive symptoms	415	Black	Mild: 12.36% Moderate: 5.21% Moderately severe: 2.02% Severe: 1.33%		PHQ-9	0–2: 27.8% 3–7: 24.8% 8–15: 23.5% ≥ 16: 23.9%	Unspecified	75
Briganti et al. [85]	2018	Brazil	Cross-sectional	Depressive symptoms	29	Asian	Mild: 12.93% Moderate: 4.38% Moderately severe: 2.29% Severe: 1.00%		PHQ-9	0–2: 27.8% 3–7: 24.8% 8–15: 23.5% ≥ 16: 23.9%	Unspecified	75
Briganti et al. [85]	2018	Brazil	Cross-sectional	Depressive symptoms	1,608	Brown	Mild: 13.56% Moderate: 5.06% Moderately severe: 2.05% Severe: 1.11%		PHQ-9	0–2: 27.8% 3–7: 24.8% 8–15: 23.5% ≥ 16: 23.9%	Unspecified	75
Briganti et al. [85]	2018	Brazil	Cross-sectional	Depressive symptoms	27	Indigenous	Mild: 22.24% Moderate: 5.16% Moderately severe: 2.82% Severe: 0.13%		PHQ-9	0–2: 27.8% 3–7: 24.8% 8–15: 23.5% ≥ 16: 23.9%	Unspecified	75
Walls et al. [86]	2014	USA	Cross-sectional	Depressive symptoms	218	American Indian	17.10%	57	PHQ-9 (≥ 10)	15	Type 2	65
Walls et al. [86]	2014	USA	Cross-sectional	Anxiety	218	American Indian	Moderate: 12.9% Severe:12%	57	BAI (≥ 16)	15	Type 2	65
Sunny et al. [87]	2019	Nepal	Cross-sectional	Depression	278	Dalit	3, 4.7% (*p* = 0.16)	54.3, 11.2	BDI-II (≥ 16)		Type 2	70
Sunny et al. [87]	2019	Nepal	Cross-sectional	Depression	278	Janjati	9, 14.3% (*p* = 0.16)	54.3, 11.2	BDI-II (≥ 16)		Type 2	70
Sunny et al. [87]	2019	Nepal	Cross-sectional	Depression	278	Madhesi	38, 60.3% (*p* = 0.16)	54.3, 11.2	BDI-II (≥ 16)		Type 2	70
Sunny et al. [87]	2019	Nepal	Cross-sectional	Depression	278	Muslin	11, 17.5% (*p* = 0.16)	54.3, 11.2	BDI-II (≥ 16)		Type 2	70
Sunny et al. [87]	2019	Nepal	Cross-sectional	Depression	278	Brahmin/Chhetri	2, 3.2% (*p* = 0.16 s	54.3, 11.2	BDI-II (≥ 16)		Type 2	70
Palta et al. [88]	2014	USA	Cohort	Depression	506	Hispanic	192, 37.9% (*p* = 0.018)	73.0, 6.5	SHORT-CARE		Type 2	Good
Palta et al. [88]	2014	USA	Cohort	Depression	95	African-American (non-Hispanic)	21, 22.1% (*p* = 0.018)	73.0, 6.5	SHORT-CARE		Type 2	Good
Palta et al. [88]	2014	USA	Cohort	Depression	4	White (non-Hispanic)	3, 75% (*p* = 0.018)	73.0, 6.5	SHORT-CARE		Type 2	Good
Golden et al. [89]	2017	USA	Cross-sectional	Depression	103	White	MinD: 23.5% MDD: 33.3% (*p* = 0.67)	Without: 61.5, 12.1 MinD: 55.4, 11.3 MDD: 57.1, 10.2	PHQ-2 (≥ 3)		Type 2	90
Golden et al. [89]	2017	USA	Cross-sectional	Depression	103	Black	MinD: 76.5% MDD: 58.3% (*p* = 0.67)	Without: 61.5, 12.1 MinD: 55.4, 11.3 MDD: 57.1, 10.2	PHQ-2 (≥ 3)		Type 2	90
Yu et al. [90]	2016	China	Cross-sectional	Depression	1123	Han	97, 8.6% (*p* = 0.53)		PHQ-9 (≥ 10)		Unspecified	75

Abbreviations are as follows: *MINI* (Mini International Neuropsychiatric Interview), *TEI* (Traumatic Events Interview), *ZDRS* (Zung Depression Rating Scale), *PHQ-9* (Patient Health Questionnaire-9), *GAD-7* (Generalised Anxiety Disorder-7), *AUDIT-C* (Alcohol Use Disorders Identification Test for Consumption), *DSM-5* (Diagnostic and Statical Manual of Mental Disorders-5), *CES-D* (Center for Epidemiological Studies-Depression Scale), *ICD-9 CM* (International Classification of Diseases, Ninth Revision, Clinical Modification), *HADS* (Hospital Anxiety and Depression Scale), *GDS-15* (Geriatric Depression Scale), *BRFSS* (Behavioral Risk Factor Surveillance Survey), VHA’s *WHEI* (Women’s Health Evaluation Initiative), *MPSS* (Modified PTSD Symptom Scale), *BDI* (Beck Depression Inventory), *PC-PTSD* (Primary Care PTSD), *BLDS* (Brief Lifetime Depression Scale), *ZSAS* (Zung Self-Rating Anxiety Scale), *Beck Anxiety Inventory* (BAI), *MinD* (minor depressive disorder), *MDD* (major depressive disorder).Grey shading indicates that the information was unavailable from the article

### Study Quality

The methodological quality of the included studies is summarised in Table 2. Overall, all studies had clearly stated objectives, used appropriate study designs aligned with their aims, and presented data and conclusions that were supported by their findings. Most studies (*n* = 37) adequately described their target populations, used validated tools for assessing mental health (*n* = 42) and diabetes-related variables, and clearly defined risk factors and outcome measures (*n* = 41). However, common methodological limitations were observed. Most studies did not justify their sample sizes (*n* = 35) and did not report efforts to address or categorise non-respondents (*n* = 39). Additionally, many studies (*n* = 27) either had response rates below 50% or failed to report response rates altogether, raising concerns regarding potential non-response bias. These limitations should be considered when interpreting the findings of the included literature.

### Meta-analysis of the Prevalence of Mental Health Disorders

Prevalence of mental health disorders among individuals with diabetes across different ethnic groups.

**Fig. 2:**
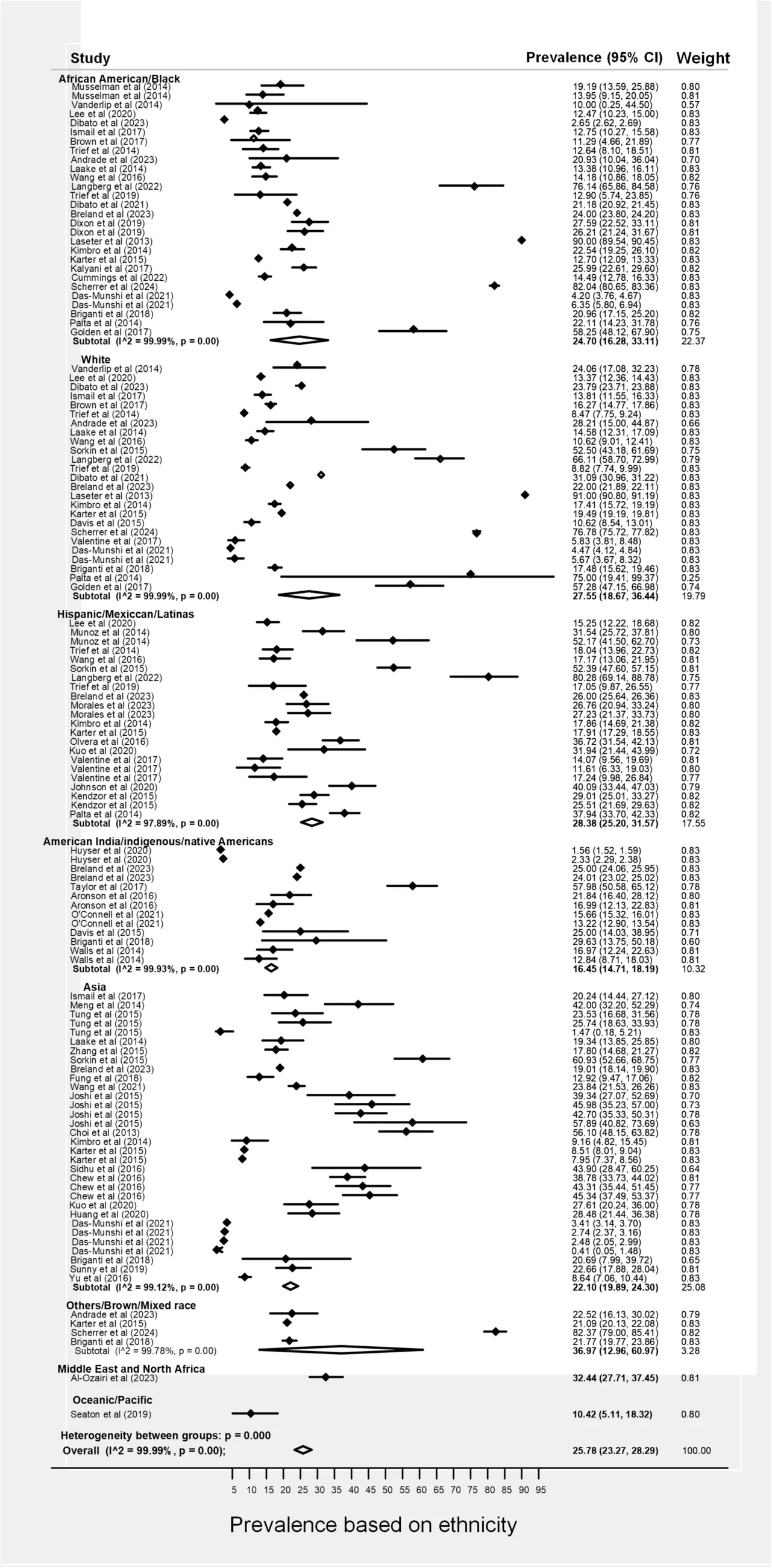
Mental health disorder (MHD) PR by ethnicity Forest Plot

Figure 2 presents a forest plot of the pooled estimated prevalence of mental health disorders among individuals with Diabetes across different ethnic groups. The estimated pooled prevalence of mental health disorders was 25.78% (95% CI: 23.27–28.29), with high heterogeneity (I^2^ = 99.9%) across studies. The prevalence of mental health disorders was highest among unspecified ethnic groups/Mixed race (36.97%, 95% CI: 12.96–60.97) followed by individuals from North Africa and the Middle East (32.44%, 95% CI: 27.71–37.45), and Hispanics or Latinos (28.36%, 95% CI: 25.20–31.57). The lowest pooled prevalence was observed among Oceanians or Pacific Islanders (10.42%, 95% CI: 5.11–18.32). Meta-regression analysis showed a significant variation in the weighted prevalence of mental health disorders across ethnic groups, with an overall *p*-value < 0.001. Meta-regression analysis identified a statistically significant interaction between study year and mental health disorder prevalence among individuals with diabetes, varying across different ethnic groups (*P* < 0.001). The funnel plot shows that the distribution of studies appears balanced, indicating little to no publication bias (see supplementary Fig. 1 for details) and the plot of log-transformed proportion of mental health disorders among individuals with diabetes by year of publication indicated a slight decrease in the proportion of mental health disorders among individuals with diabetes over the study period (see supplementary Fig. 2 for details).

### Prevalence Based on type of Mental Health Disorder

**Fig. 3:**
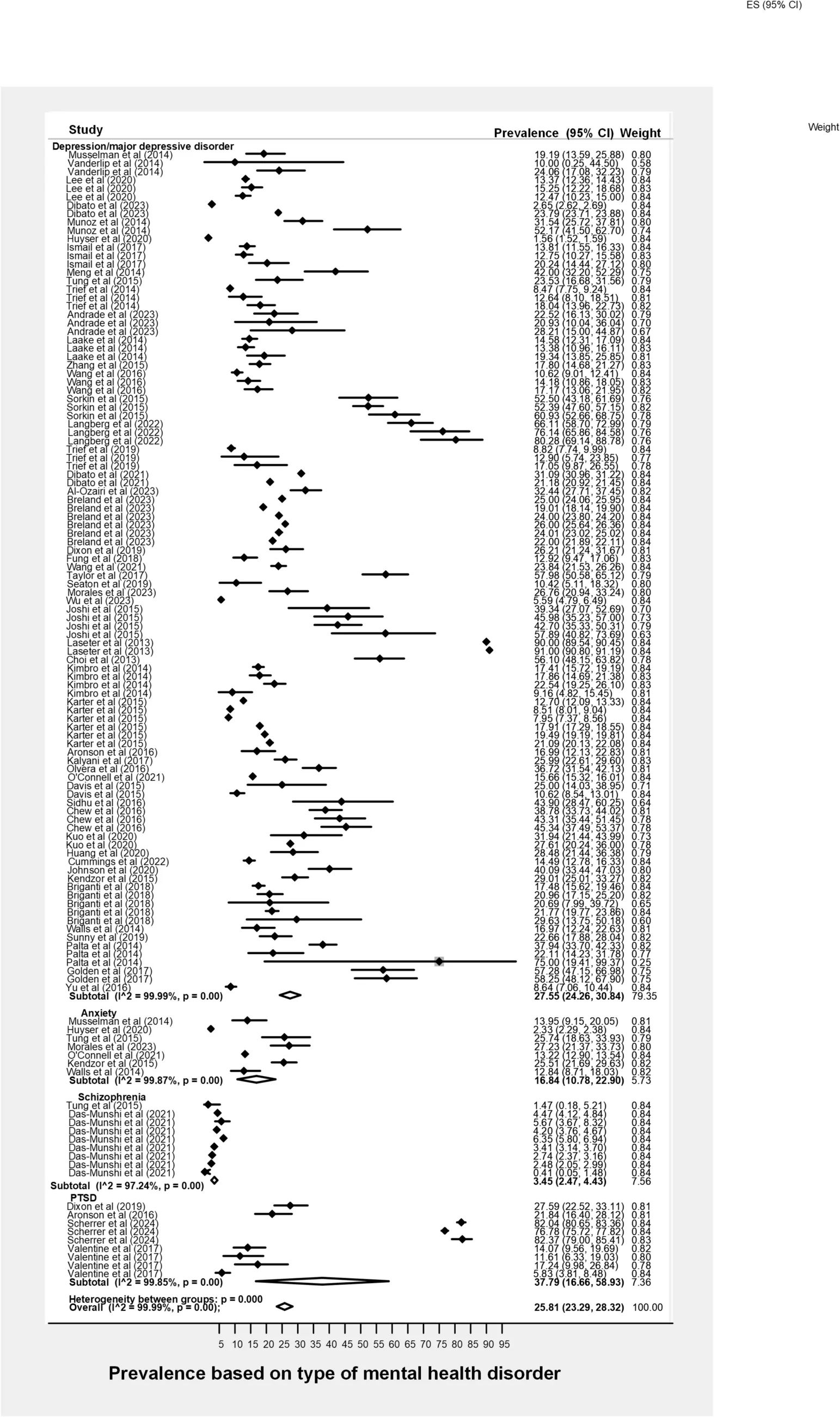
MHD PR by type of MHD Forest Plot

Figure 3 is a forest plot illustrating the pooled estimated prevalence of mental health disorders among individuals with diabetes, stratified by type of disorder. The overall pooled prevalence estimate based on type of mental health disorders was 25.81% (95% CI: 23.29–28.32) and it varied significantly across studies (I^2^ = 99.99%, *p* < 0.0001). The most prevalent mental health disorder was post-traumatic stress disorder [PTSD, 37.79%, (95% CI: 16.66–58.93)], followed by depression [27.55% (95% CI: 24.26–30.84)]. Schizophrenia was the least prevalent mental health disorde in this study, with an estimated prevalence of 3.45% (95% CI: 2.47–4.43). There was a significant interaction between type of mental health disorder and year of study (*p* < 0.001).

### Prevalence Based on the Type of Diabetes

**Fig. 4:**
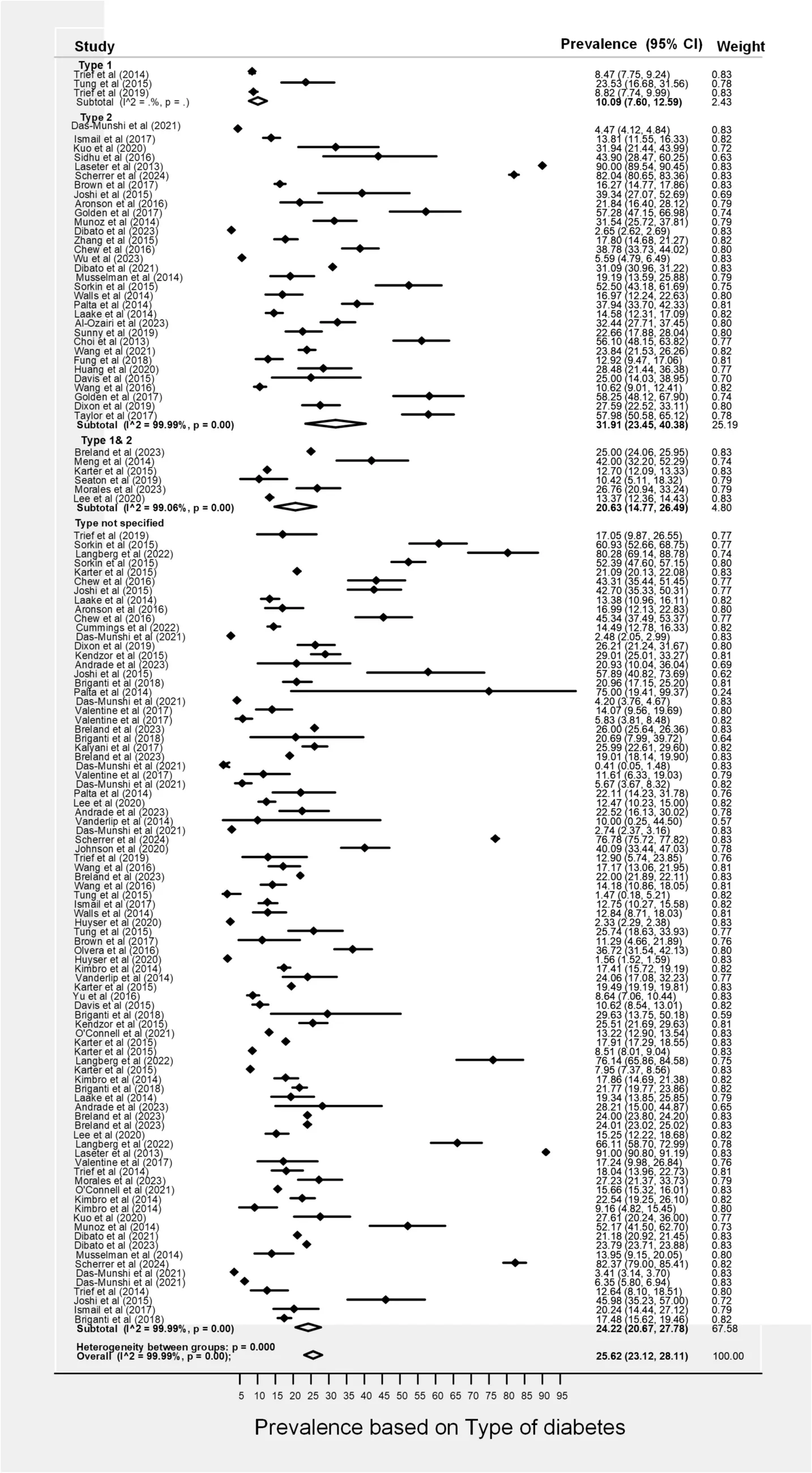
MHD PR by type of diabetes Forest Plot

Figure 4 is a forest plot illustrating the pooled estimated prevalence of mental health disorders based on the type of diabetes. The overall prevalence of mental health disorders across all Diabetes types was 25.62% (95% CI: 23.12–28.11), with high heterogeneity observed (I^2^ = 99.9%). The highest prevalence was found in individuals with type 2 diabetes (31.91%, 95% CI: 23.45–40.38), followed by unspecified type of diabetes (24.22%, 95% CI: 20.67–27.78) and the lowest prevalence was among people with type 1 diabetes (10.09%, 95% CI: 7.60–12.59). Meta-regression analysis showed a significant variation in the weighted prevalence of mental health disorders based on type of diabetes, with an overall *p*-value < 0.001. A statistically significant interaction was observed between the year of study and the type of diabetes (*P* < 0.001).

### Prevalence Based on the Income Level of Countries

**Fig. 5:**
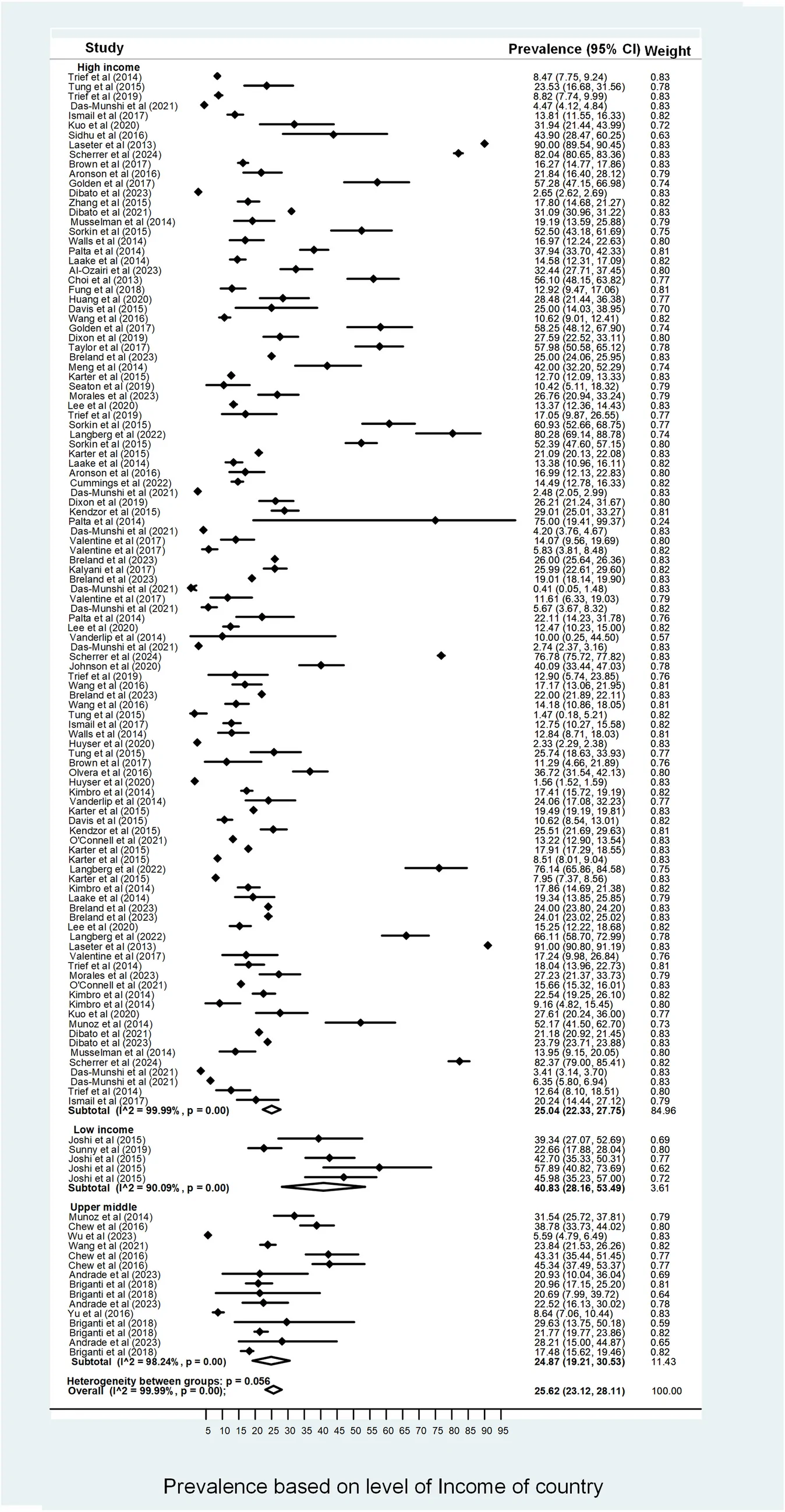
MHD PR by level of income Forest Plot

The forest plot of the estimated pooled prevalence of mental health disorders based on the country’s level of income is presented in Fig. 5. In people with diabetes, there overall pooled prevalence 25.62% (95% CI: 23.12–28.11) with high heterogeneity (I^2^ = 99.9%). The low-income countries had the highest prevalence (40.63%, 95% CI: 28.16–53–49) while the upper-middle-income countries had the lowest prevalence of mental health disorder (24.87%, 95% CI: 19.30.53). A statistically significant interaction was found between the year of study and the level of income of the country (*P* < 0.001).

### Prevalence Based on Duration of Diabetes

**Fig. 6:**
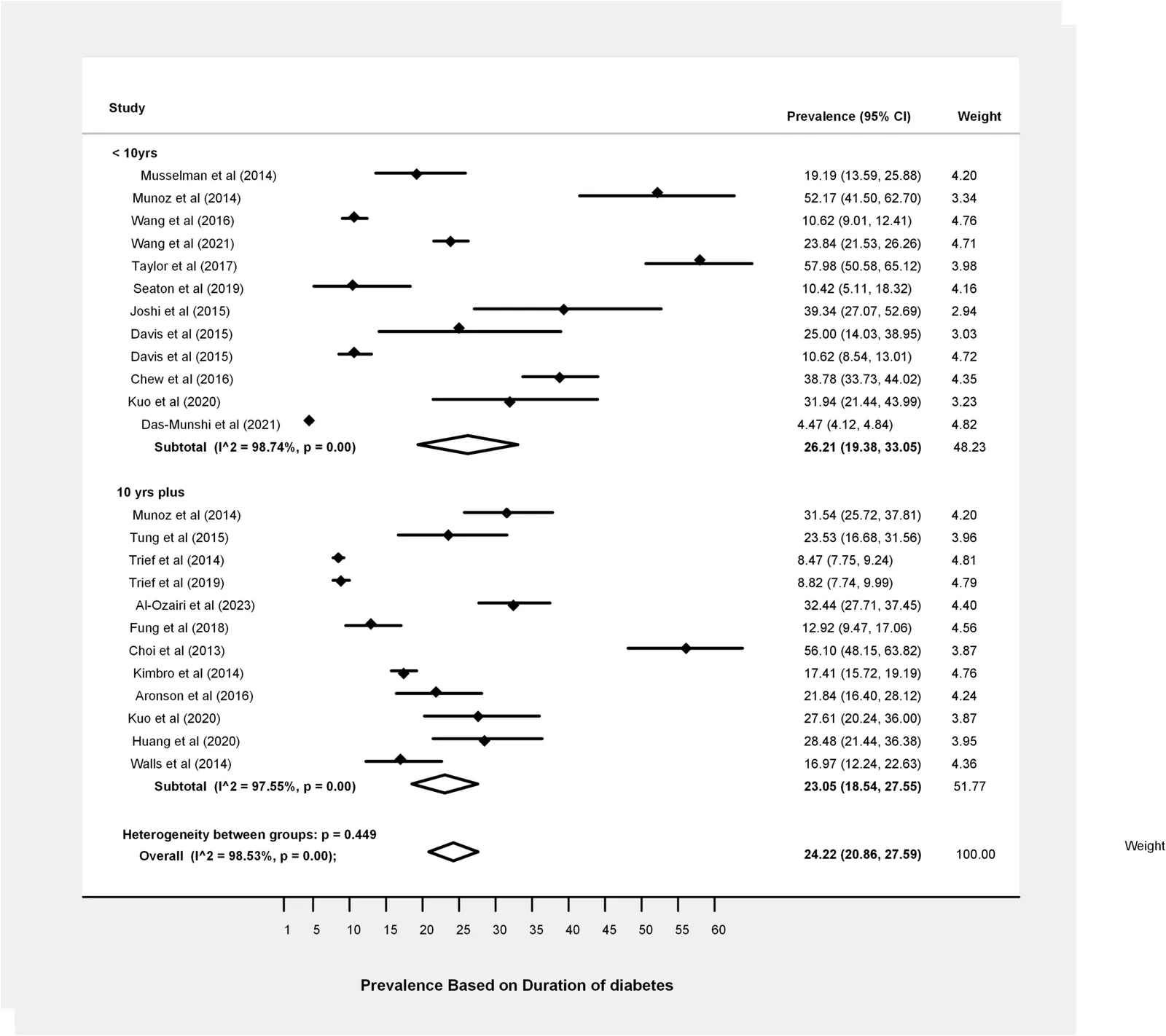
Mental health disorder Prevalence by duration of diabetes

The forest plot of the estimated pooled prevalence of mental health disorders based on duration of diabetes is presented in Fig. 6. It shows that the pooled prevalence of mental health disorders based on the duration of Diabetes was 24.22% (95% CI: 20.86–27.59), and this varied significantly with the duration of diabetes, and there was also a high heterogeneity (I^2^ = 99.9%). Although the prevalence was slightly higher in adults who have had diabetes for less than 10 years (26.21%, 95% CI: 19.38–33.05) compared with those who have had diabetes for more than 10 years (23.05%, 95% CI: 18.54–27.55), the confidence intervals were overlapping suggesting no significant difference. The interaction between year of study and duration of diabetes was statistically significant (*p* < 0.001).

### Prevalence Based on the Mental Health Disorder Diagnostic Method

**Fig. 7:**
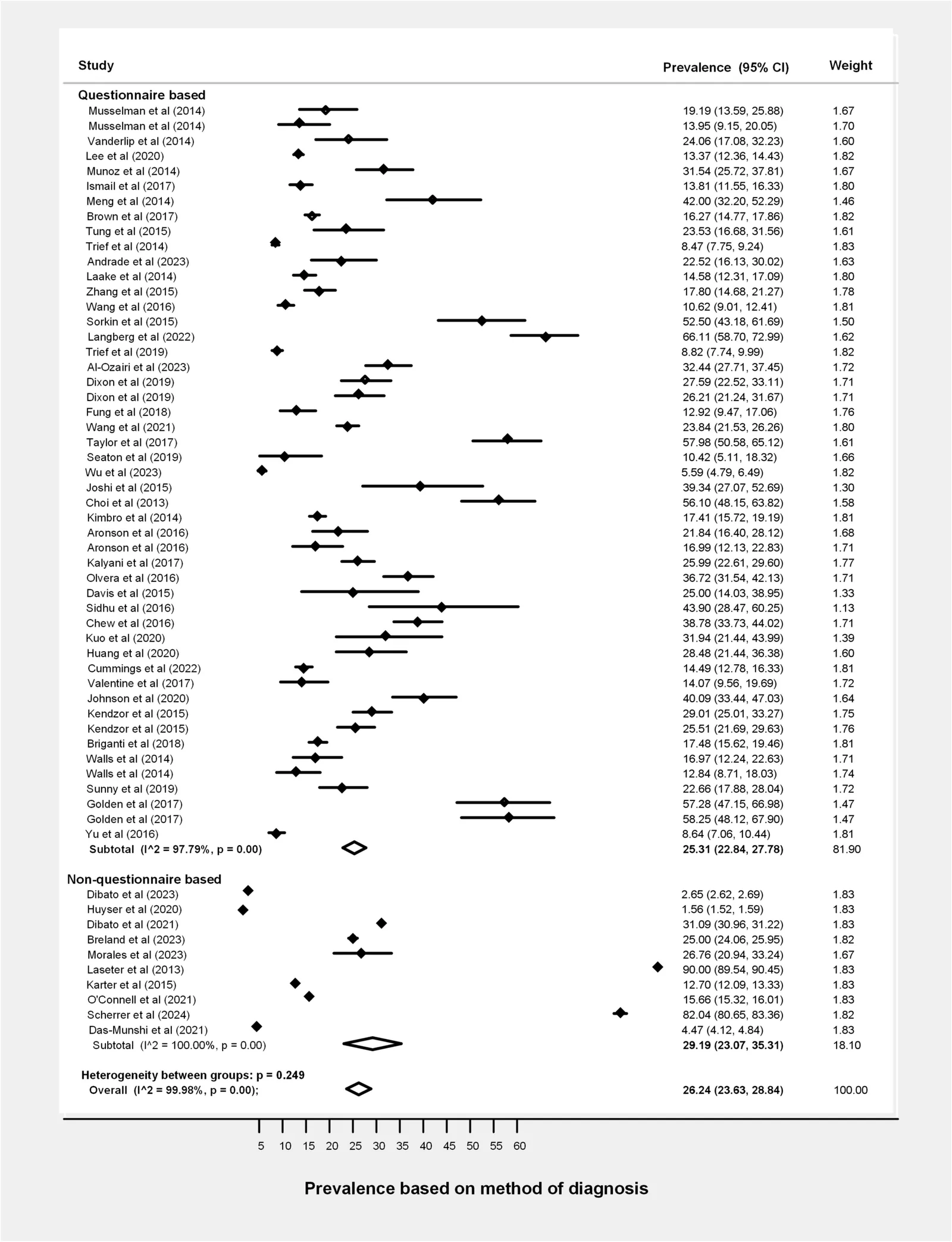
MHD PR by method of diagnosis Forest Plot

Figure 7 presents the forest plot of the estimated pooled prevalence of mental health disorders based on diagnostic method. The estimated pooled prevalence of mental health disorders based on the diagnosis method was 26.24% (23.63–28.84) with high heterogeneity (I^2^ = 99.98%, *p* = 0.00) across studies, which was statistically significant. The prevalence was comparable when determined using a clinical assessments, medical records, or a diagnostic approach (29.19%, 95% CI: 23.07–35.31) than standardized self-report questionnaires or screening tool (25.31%, 95% CI: 22.84–27.78; *p* = 0.249). Meta-regression analysis revealed a statistically significant interaction between the year of study and mental health disorder diagnostic method (*p* < 0.001).

### Prevalence Based on Indigeneity

**Fig. 8:**
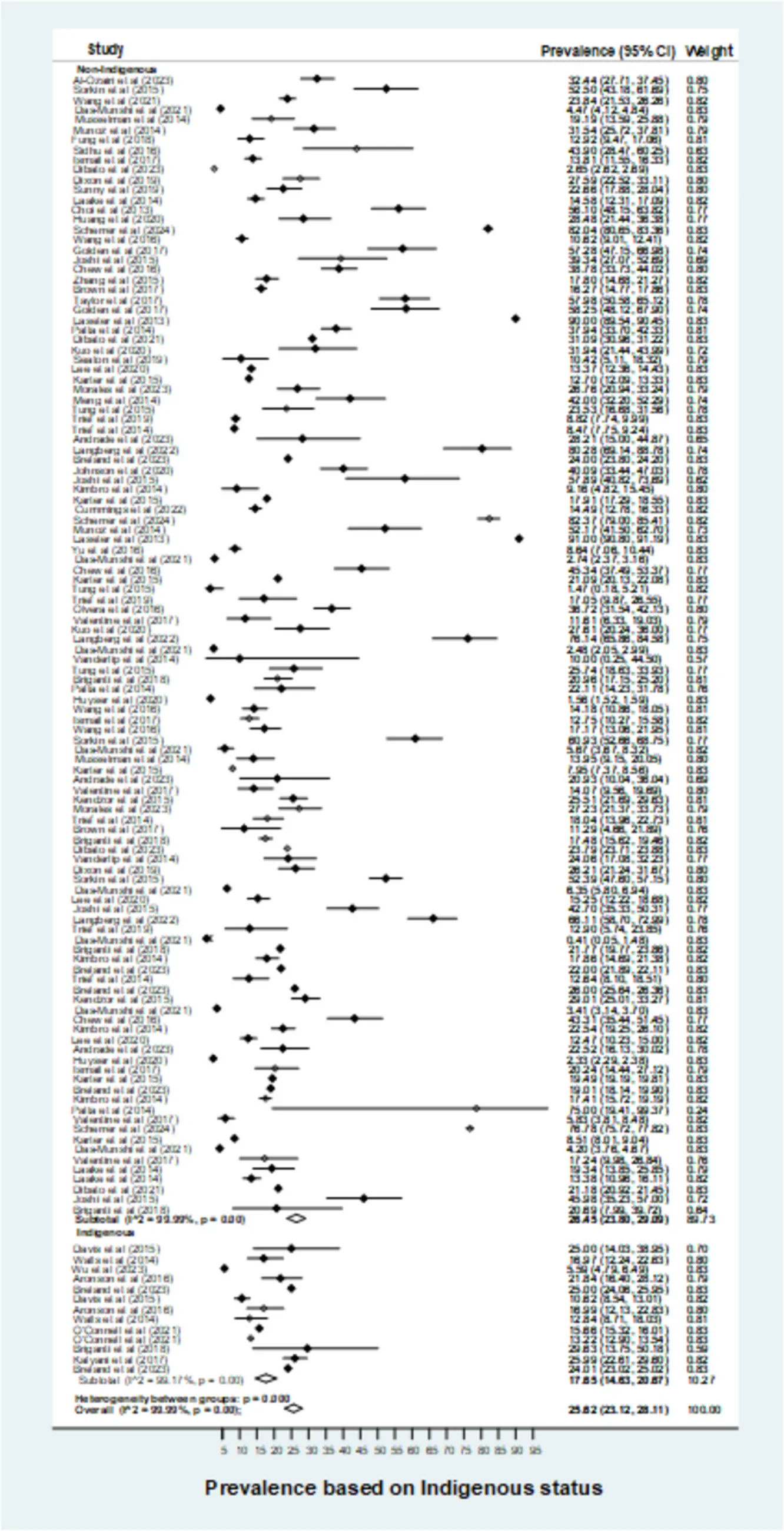
MHD PR by Indigenous status

Figure 8 is a forest plot illustrating the estimated pooled prevalence of mental health disorders by Indigenous status. The estimated pooled prevalence of mental health disorders based on indigeneity was 25.62% (95% CI: 23.12–28.11), with high heterogeneity (I^2^ = 99.9%). The prevalence of mental health disorders was higher in non-indigenous people (26.45%, 95% CI: 23.80–29.09) than in indigenous people (17.65%, 95% CI: 14.63–20.67) with diabetes, with a significant interaction between year of study and indigeneous status (*p* < 0.001).

### Pooled Mean Age

**Fig. 9:**
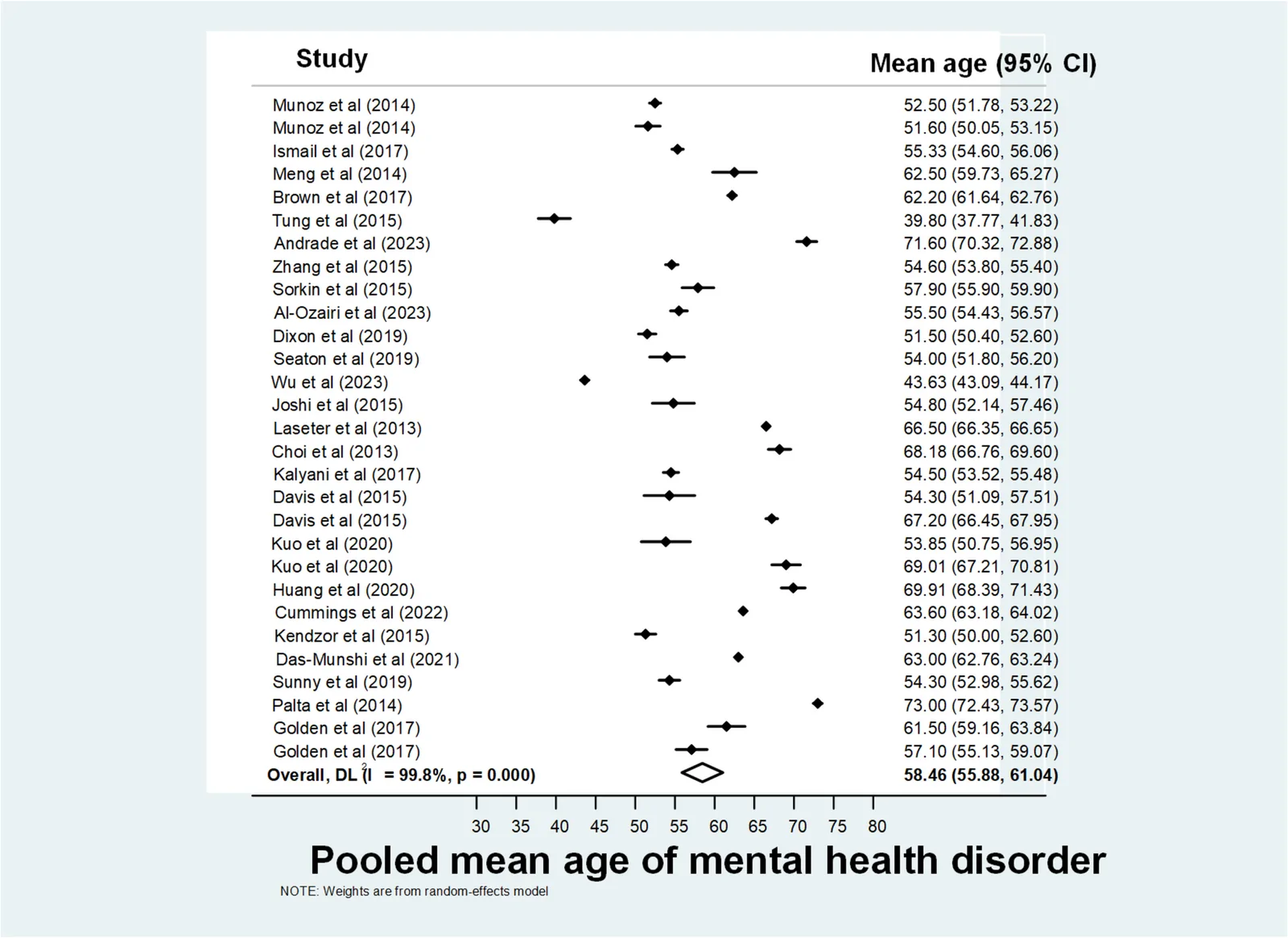
Pooled mean age of participants with mental health disroder in the studies

Figure 9 presents the forest plot illustrating the pooled mean age of individuals with mental health disorders across included studies. The overall pooled mean age was 58.46 years (95% CI: 55.88–61.04), with substantial heterogeneity observed (I^2^ = 99.8%), indicating considerable variability in mean age estimates between studies. Most individuals with mental health disorders were aged over 50 years. Meta-regression analysis identified the highest estimated mean age as 73.00 years (95% CI: 72.43–73.57) and the lowest as 39.80 years (95% CI: 37.77–41.83).

### Subgroup Analyses

To explore potential sources of the high heterogeneity observed in the pooled estimates (I^2^ > 99%), we conducted subgroup analyses based on ethnicity, mental health diagnostic method, and national income level. Stratification by diagnostic method showed a slight reduction in heterogeneity among studies that employed questionnaires or standardized tools (I^2^ = 97.79%). In the ethnicity-based subgroup analysis, studies conducted among Hispanic populations also exhibited marginally lower heterogeneity (I^2^ = 97.89%) compared to other ethnic subgroups. When stratified by national income level, the heterogeneity observed among studies from the low-income countries was slightly lower (I^2^ = 90.09%), with greater variability between studies from high-income (I^2^ = 99.99%) and upper-middle-income countries (I^2^ = 98.24%). These findings suggest that differences in population characteristics, assessment tools, and health system contexts may partially explain the substantial between-study variability.

## Discussion

This is the first known attempt to provide a pooled estimate of the prevalence of mental health disorders among individuals with diabetes across diverse ethnic groups. The findings reveal a notably high overall pooled prevalence of mental health disorders (25.78%) in people with diabetes. The marked variation in the prevalence of mental health disorders observed across different ethnic and socioeconomic groups, suggests there are differing vulnerabilities. Aside from people with unspecified ethnic groups, those from the Middle East and North Africa (MENA) region had a high prevalence of mental health disorders, while the lowest prevalence was noted among individuals of Oceanian or Pacific Island descent. Prevalence was also higher in low-income countries compared to upper-middle-income countries, with further subgroup analyses revealing notable trends**:** mental health disorders were more prevalent among those aged over 50 years, individuals with type 2 diabetes, those diagnosed with diabetes for less than 10 years, and individuals diagnosed using questionnaire-based methods. Additionally, non-indigenous populations were found to have a higher prevalence of mental health disorders compared to Indigenous groups. Interestingly, post-traumatic stress disorder (PTSD) emerged as the most common mental health disorder, followed by depression, with schizophrenia being the least common mental health condition in people with diabetes.

The high heterogeneity recorded in this study may be attributed to the cultural differences in mental health awareness, stigma, and reporting. In addition, the variation in diagnostic instruments, ranging from clinical interviews to self-reports, with differing sensitivities, socioeconomic disparities, and healthcare access differences across countries may contribute in part to the high heterogeneity. These factors likely contributed to inconsistent prevalence rates of mental health disorders among individuals with diabetes. Understanding these sources is crucial for contextualizing our findings and underscores the need for standardized assessment and reporting across diverse populations.

The high prevalence of mental health disorders in the current study reflects a critical comorbidity that significantly affects diabetes management and quality of life [91, 92]. The fact that in the present study, significantly more individuals with diabetes may also experience mental health challenges, compared with the 19.5% reported in a large multinational study by Lin and Von Korff [93] highlights the critical need for integrated care approaches. Although the differences in findings could be attributed to the types of disorders assessed, methodological differences, and population characteristics of both study participants, many prior studies [5, 93] have also reported lower prevalence and have focused solely on depression or anxiety, making it difficult to directly compare our study with theirs.

The profound ethnic-based variations in the prevalence of mental health disorders observed in the current study may reflect differences in cultural perceptions of mental illness, access to mental health services, socio-economic status, and exposure to structural inequities [15]. The high prevalence recorded among individuals from the MENA region aligns with previous studies and may be linked to the high burden of diabetes and prediabetes reported in this region [94–96]. However, our results differ from the findings of Lin and Von Korff [93], which reported higher mental health disorder prevalence among individuals of European descent. This discrepancy may reflect underreporting of mental health symptoms in certain ethnic groups, such as those from Oceania and Sub-Saharan Africa, where stigma around mental health may influence diagnosis and reporting [97, 98]. The high prevalence among mixed or unspecified ethnic groups may also highlight data limitations or population-level heterogeneity that warrants further disaggregation.

Cross-country and ethnic differences observed in our study may be influenced by a range of cultural and contextual factors. Mental health is conceptualized and expressed differently across cultures, which may affect how symptoms are reported, recognized, and diagnosed. For instance, individuals from some Asian and African cultures may be more likely to somatize psychological distress, presenting with physical symptoms rather than emotional complaints, which can lead to underdiagnosis of mental disorders [99]. Additionally, stigma surrounding mental illness remains deeply rooted in many societies, especially among certain ethnic minorities, and this can discourage individuals from seeking help or participating in mental health screenings [100]. These cultural factors, coupled with disparities in healthcare access and the availability of culturally competent mental health services, may partially explain the variation in prevalence rates across countries and ethnic groups observed in our review. Understanding and addressing these cultural dynamics is crucial for developing appropriate screening tools and interventions tailored to diverse populations.

The present findings contrast with the broader literature, where depression is typically reported as the most prevalent mental health disorder among individuals with diabetes [93, 95, 101–103]. PTSD is known to have profound effects on physical health through mechanisms such as increased inflammation and altered metabolic function, which could potentially exacerbate the management of Type 2 diabetes. The prominence of PTSD in this review must be interpreted with caution due to the limited number of studies assessing this disorder. All four PTSD studies included in our analysis were conducted in the United States and focused on trauma-exposed and socially marginalized populations, including African American women [58], American Indian adults [70], military veterans [80] and Latinos [81]. These populations experience higher exposure to trauma, chronic stress, and systemic disparities, which may explain the elevated prevalence of PTSD observed in our findings. Moreover, all studies used validated diagnostic tools based on DSM criteria, potentially contributing to more accurate case detection compared to studies on depression, which often relied on screening instruments. PTSD is known to have profound effects on physical health through mechanisms such as increased systemic inflammation, dysregulated hypothalamic–pituitary–adrenal (HPA) axis activity, and altered metabolic function. These biological changes may worsen glycemic control and complicate the management of type 2 diabetes. Thus, while the elevated PTSD prevalence observed may not be generalizable to the broader diabetic population, it highlights the need for integrated care approaches that account for trauma history, particularly in high-risk populations. These findings underscore the importance of screening for PTSD in individuals with diabetes, particularly among underserved and trauma-affected communities. Future research should explore the prevalence and impact of PTSD in more diverse and representative populations to better understand its role in the mental health burden of diabetes.

Although depression was not the most prevalent in our study, the rate was higher than that reported in studies from the USA, Palestine, and Jordan [17, 97, 98, 104, 105], but lower than the 38% reported in a systematic review from India [94]. These discrepancies may reflect differences in diagnostic tools, such as the use of the PHQ-8 in our study versus the BDI-II or CES-D used in other studies, which may lead to variations in prevalence estimates [101]. Furthermore, the diverse methodological approaches employed in these studies, such as differences in sample characteristics and the inclusion of various mental health disorders, likely contribute to the observed heterogeneity in the findings. Conversely, schizophrenia was the least prevalent disorder**,** a finding consistent with global data [101, 103, 106]. Its low prevalence may reflect rarity or potential underdiagnosis of the condition among individuals with diabetes.

Consistent with our study findings, a study in Jordan [104] recorded a higher prevalence of mental health disorders among individuals with type 2 diabetes (20.1%) than those with type 1 diabetes (16.2%). In contrast, some studies recorded a higher prevalence of mental health disorders in type 1 than type 2 diabetes [17, 95]. The higher mental health burden in Type 2 Diabetes may be explained by factors such as later age of onset, greater risk of diabetes-related complications, and socioeconomic burden. On the other hand, individuals with Type 1 diabetes may benefit from earlier diagnosis and structured care, which might help mitigate the mental health burden.

Our findings suggest higher prevalence rates when mental health disorders were assessed using non-questionnaire-based diagnostic methods (29.19%) compared to questionnaire-based tools (25.31%), though the difference was not statistically significant. This may indicate potential underestimation in self-reported data or overestimation in clinical settings, raising concerns regarding measurement bias and diagnostic standardization [28, 101]. Self-reported instruments such as the PHQ-9, while widely used for their ease of administration and scalability, may be influenced by factors such as literacy levels, cultural expression of psychological symptoms, and social desirability bias, particularly in ethnically diverse or low-resource populations. Conversely, clinician-administered interviews offer greater diagnostic precision but are more resource-intensive and less accessible in many settings. These methodological differences may contribute to variability in prevalence estimates across studies. To improve comparability and diagnostic accuracy, future studies should aim to validate screening tools across different population subgroups (e.g., ethnicity, diabetes type), and where possible, combine self-report measures with structured clinical assessments. Establishing standardised, contextually appropriate diagnostic protocols would help mitigate the impact of methodological heterogeneity and support more accurate estimates of mental health burden in people with diabetes.

The higher prevalence of mental health disorders in low-income countries may be explained by the weaker health systems, limited access to mental health services, and greater social stigma in these countries [96, 107]. Additionally, individuals in these settings are often exposed to psychosocial stressors such as economic instability, violence, and limited access to education and employment, all of which are known contributors to poor mental health. However, this finding should be interpreted with caution, as there were only two studies from a single country, Nepal, which is a low-income country in the analysis [65, 87]. The studies, conducted in urban clinical settings, reported a high prevalence of depressive symptoms among individuals with type 2 Diabetes and contributed a combined sample size of 1,120 participants. The limited number and geographical distribution of studies from low-income settings may introduce selection bias and restrict generalizability. Despite this, the elevated prevalence observed underscores the urgent need for more population-based research across diverse low-income regions, particularly in Africa and South Asia. These findings highlight the importance of mental health awareness and the integration of mental health care into diabetes management, especially in low-resource settings. Effective strategies may include collaboration between primary care providers and mental health specialists, culturally adapted self-management programs, and chronic disease care continuity.

Although studies on Indigenous populations remain limited, our study found that the pooled prevalence of mental health disorders was lower among US Indigenous individuals with diabetes (17.65%) compared to their non-Indigenous counterparts (26.45%). This finding contrasts with existing literature, particularly from the United States, which has consistently reported a higher burden of both chronic illness and mental health conditions among Indigenous populations [91, 92, 108]. One possible explanation for this discrepancy is underdiagnosis or underreporting among Indigenous populations, potentially due to cultural differences in the expression of psychological distress, limited access to culturally safe mental health services, or the use of screening tools not validated for Indigenous contexts. However, we note that our study did not directly assess diagnostic methods or health system resource allocation, and therefore, these explanations remain speculative. The relatively small number of studies involving Indigenous participants also limits the generalisability of our findings. Nevertheless, our results are consistent with prior evidence suggesting the potential under-detection of mental health disorders in some Indigenous populations [86]. These findings highlight the importance of strengthening culturally appropriate mental health screening and care pathways, as well as the need for more robust research to understand the true burden and presentation of mental health conditions among Indigenous individuals across different regions, particularly in countries such as Canada and Australia.

Although previous studies from Palestine and Jordan [98, 104] did not find a significant association between diabetes duration and mental health outcomes; our study is consistent with the previous study and showed that individuals with diabetes less than 10 years had a similar prevalence of mental health disorder compared with those who had diabetes much longer. The slight increase in mental health disorder prevalence among non-indigenous people may reflect heightened psychological distress following diagnosis, uncertainty about disease progression, and challenges associated with early lifestyle adjustments. The pooled prevalence stratified by mean age was also substantial (58.46%), and meta-regression showed a statistically significant temporal decline, with the highest prevalence observed in 2014 and the lowest in 2017. These temporal patterns may reflect improvements in diabetes management or mental health screening over time.

On the other hand, the higher prevalence of mental health disorders observed among individuals with diabetes aged 50 years and above in the current study likely reflects the cumulative burden of diabetes-related complications, longer disease duration, and age-associated psychosocial stressors. Factors such as chronic pain, functional impairment, social isolation, and cognitive decline may contribute to increased vulnerability to depression and anxiety in older adults with diabetes. More population-based studies are needed to be conducted to confirm our findings.

### Implications of the Study

The findings of this study reveal a substantial prevalence of mental health disorders among individuals with diabetes, with notable differences across ethnic, geographic, and socioeconomic contexts. This underscores the importance of integrating mental health screening and support into diabetes care, particularly in low-income settings and among high-risk groups such as Indigenous populations and individuals with Type 2 diabetes. The prominence of PTSD and depression in this population suggests a need to move beyond the traditional focus on depression alone and include a broader spectrum of mental health conditions in clinical assessments. Furthermore, the results highlight potential systemic inequities in access to mental health care, diagnosis, and reporting, especially in underrepresented and marginalized populations.

### Recommendations

Healthcare systems should adopt integrated models that combine mental health services with diabetes management, particularly in primary care settings. Standardized and culturally sensitive screening tools should be used routinely in diabetes care to detect mental health disorders early, especially PTSD and depression, which emerged as the most prevalent disorders in this study.

Tailored interventions should be developed for high-risk populations such as Indigenous communities, individuals with Type 2 diabetes, and those in low-income countries. Training of healthcare providers, especially in low-resource settings, is essential to improve the identification and management of comorbid mental health disorders. Future studies should prioritize underrepresented groups and use standardized diagnostic tools to allow for comparability and better inform public health strategies.

### Strengths and Limitations of Study

This is the first known study to systematically quantify the pooled prevalence of mental health disorders among individuals with diabetes across diverse ethnic and economic groups. The inclusion of a wide range of mental health disorders (e.g., PTSD, anxiety, depression, schizophrenia) provides a more holistic picture of the mental health burden in diabetes. Stratified analyses by diabetes type, diagnostic method, income level, and indigeneity offer valuable insights into high-risk groups and contextual factors. The findings have important implications for healthcare planning and policy, especially in promoting equity in chronic disease and mental health care delivery. Despite the strengths, some limitations need to be acknowledged. A key limitation in this study was the substantial heterogeneity (I^2^ > 99%) in the prevalence estimates observed within and between ethnic groups. Although the subgroup analyses based on ethnicity, diagnostic tools, and national income levels showed slightly lower heterogeneity in certain ethnic groups compared with the others, for example, studies using standardized tools or questionnaire-based based had lower within-group heterogeneity (I^2^ = 97.79%) compared to non-questionnaire-based studies, the study was unable to further classify the ethnic groups into subgroups as this was not often reported in the reviewed studies.

Also, the inclusion of only English-language articles may introduce language bias. Most studies were from high-income countries, potentially underrepresenting low- and middle-income settings. Populations from Oceania, Sub-Saharan Africa, and Indigenous communities outside the USA were underrepresented, potentially limiting the generalizability of findings to these groups. Variability in the diagnostic tools used across studies (e.g., PHQ-9, CES-D, PC-PTSD, self-report) may have influenced prevalence estimates and complicated direct comparisons. Most included studies were cross-sectional, which limits causal inferences between diabetes and mental health disorders. Cultural stigma and mistrust of healthcare systems may have led to underreporting of mental health symptoms, particularly in Indigenous and ethnic minority populations. Some studies did not clearly define ethnicity or indigeneity, and broad groupings such as "unspecified ethnicity" which could have masked important within-group differences.

## Conclusion

There was a high prevalence of mental health disorders among individuals with diabetes in different ethnic groups, emphasizing the interplay between biological, psychosocial, and systemic factors. The results point to an urgent need for mental health integration in diabetes care, particularly for vulnerable populations such as those in low-income regions and Indigenous communities. Addressing these disparities requires a coordinated effort involving healthcare providers, policymakers, and researchers. Standardized diagnostic practices, culturally sensitive care models, and targeted interventions are essential to improving outcomes and achieving equity in the management of diabetes and comorbid mental health conditions.

## Supplementary Information

Below is the link to the electronic supplementary material.

Supplementary file1 (DOCX 495 KB)

## Data Availability

The data used to support the findings of this study are made available from the corresponding author upon reasonable request.
