# Supplementary material for: Ethnic Differences in the Prevalence of Mental Health Disorders Among Individuals with Diabetes: A Systematic Review and Meta-analysis
Source: J Racial Ethn Health Disparities. 2025 Nov 10;13(6):5168–207. doi: 10.1007/s40615-025-02656-7 (PMC13754959; doi:10.1007/s40615-025-02656-7)
Supplement: Supplementary file 1 [file 40615_2025_2656_MOESM1_ESM.docx]

Appendices

Appendix 1

**MEDLINE**

((diabetes mellitus/ or diabetes mellitus, experimental/ or diabetes mellitus, type 1/ or diabetes mellitus, type 2/ or diabetes mellitus, lipoatrophic/ or latent autoimmune diabetes in adults/ or prediabetic state/) OR (diabet* or prediabet* or “impaired glucose tolerance” or “impaired fasting glucose” or T2D* or T1D*).ti,ab.) AND (exp Adult/ OR (adult* or middle aged or aged or person* or people or patient*).ti,ab.) AND ((mental disorders/ or exp anxiety disorders/ or exp dissociative disorders/ or exp "feeding and eating disorders"/ or exp mood disorders/ or exp paraphilic disorders/ or exp personality disorders/ or exp "schizophrenia spectrum and other psychotic disorders"/ or exp "trauma and stressor related disorders"/ or exp Mental Health/ or depersonalization/ or depression/) OR (“mental illness” or “mental health disorder” or “mental problem” or “mental disorder” or “mental disease” or psychiatric or “psychotic disorder” or psychosis or psychotrauma or “mood disorder” or schizophrenia or “delusional disorder” or depressi* or “bipolar disorder” or anxiety or “panic disorder” or “obsessive-compulsive disorder” or “post-traumatic stress disorder” or “eating disorder” or “anorexia nervosa” or “bulimia nervosa” or “binge-eating disorder” or “personality disorder”).ti,ab.) AND ((exp "health disparate minority and vulnerable populations"/ or exp population groups/) OR (ethnic* or racial* or race or cultur* or minority* or immigrant* or migrant* or native or Indigenous or Aboriginal or “First Nation*” or “African American” or “American Indian” or “Mexican American” or Caucasian or Maori or Hispanic or Latino or Caribbean or “Middle Eastern” or Chinese).ti,ab.) AND yr=”2014-2024”

**Embase**

((exp diabetes mellitus/ or exp impaired glucose tolerance/ or exp diabetic patient) OR (diabet* or prediabet* or “impaired glucose tolerance” or “impaired fasting glucose” or T1D* or T2D*).ti,ab.) AND ((adult/ or exp aged/ or exp middle aged/ or exp young adult/) OR (adult* or middle aged or aged or person* or people or patient*).ti,ab.) AND (exp mental health/ or mental disease/ or exp anxiety disorder/ or behavior disorder/ or exp mood disorder/ or exp personality disorder/ or exp psychosis/ or exp schizophrenia spectrum disorder/) OR (“mental illness” or “mental health disorder” or “mental problem” or “mental disorder” or psychiatric or “psychotic disorder” or psychosis or “mood disorder” or schizophrenia or “delusional disorder” or depressi* or “bipolar disorder” or anxiety or “panic disorder” or “obsessive-compulsive disorder” or “post-traumatic stress disorder” or “eating disorder” or “anorexia nervosa” or “bulimia nervosa” or “binge-eating disorder” or “personality disorder”).ti,ab.) AND (exp ethnicity/ or exp ethnic group/ or exp minority group/) OR (ethnic* or racial* or race or cultur* or minority* or immigrant* or migrant* or native or Indigenous or Aboriginal or “First Nation*” or “African American” or “American Indian” or “Mexican American” or Caucasian or Maori or Hispanic or Latino or Caribbean or “Middle Eastern” or Chinese).ti,ab.) AND yr=”2014-2024”

**APA PsycInfo**

((DE "Diabetes Mellitus" OR DE "Type 1 Diabetes" OR DE "Type 2 Diabetes" OR DE "Type 1 Diabetes" OR DE "Diabetes") OR (diabet* or prediabet* or “impaired glucose tolerance” or “impaired fasting glucose” or T2D* or T1D*).ti,ab.) AND (AG "adulthood (18 yrs & older)") or (AG "aged (65 yrs & older)") or (AG "middle age (40-64 yrs)") or (AG "thirties (30-39 yrs)") or (AG "very old (85 yrs & older)") or (AG "young adulthood (18-29 yrs)") OR (TI (adult* or middle aged or aged or person* or people or patient*) OR AB (adult* or middle aged or aged or person* or people or patient*)) AND ((DE "Affective Disorders" OR DE "Major Depression" OR DE "Persistent Depressive Disorder" OR DE "Generalized Anxiety Disorder" OR DE "Panic Disorder" OR DE "Bipolar I Disorder" OR DE "Bipolar II Disorder" OR DE "Chronic Mental Illness" OR DE "Chronic Psychosis" OR DE "Anorexia Nervosa" OR DE "Binge Eating Disorder" OR DE "Bulimia" OR DE "Feeding Disorders" OR DE "Obsessive Compulsive Disorder" OR DE "Psychosis" OR DE "Serious Mental Illness" OR DE "Acute Stress Disorder" OR DE "Posttraumatic Stress Disorder" OR DE "Complex PTSD" OR DE "Mental Health Disparities") OR (TI (“mental illness” or “mental health disorder” or “mental problem” or “mental disorder” or “mental disease” or psychiatric or “psychotic disorder” or psychosis or psychotrauma or “mood disorder” or schizophrenia or “delusional disorder” or depressi* or “bipolar disorder” or anxiety or “panic disorder” or “obsessive-compulsive disorder” or “post-traumatic stress disorder” or “eating disorder” or “anorexia nervosa” or “bulimia nervosa” or “binge-eating disorder” or “personality disorder”).ti,ab.) AND ((DE "Racial and Ethnic Groups" OR DE "African Cultural Groups" OR DE "Asians" OR DE "Black People" OR DE "European Cultural Groups" OR DE "Indigenous Populations" OR DE "Latinos/Latinas" OR DE "Middle Eastern and North African Cultural Groups" OR DE "Multiracial" OR DE "North American Cultural Groups" OR DE "Oceanian Cultural Groups" OR DE "People of Color" OR DE "Romanies" OR DE "South American Cultural Groups" OR DE "White People" OR DE "Majority Groups" OR DE "Marginalized Groups" OR DE "Minority Groups") OR (ethnic* or racial* or race or cultur* or minority* or immigrant* or migrant* or native or Indigenous or Aboriginal or “First Nation*” or “African American” or “American Indian” or “Mexican American” or Black or White or Caucasian or European or Asian or Maori or Hispanic or Latino or Caribbean or “Middle Eastern” or Chinese).ti,ab.) AND (Year: “2014-2024”)

**CINAHL**

((MH "Diabetes Mellitus") OR (MH "Diabetes Mellitus, Type 1+") OR (MH "Diabetes Mellitus, Type 2") OR (MH "Prediabetic State")) OR (diabet* or prediabet* or “impaired glucose tolerance” or “impaired fasting glucose” or T1D* or T2D*).ti,ab.) AND (((MH "Adult") OR (MH "Aged") OR (MH "Middle Age") OR (MH "Young Adult")) OR (adult* or middle aged or aged or person* or people or patient*).ti,ab.) AND (((MH "Mental Disorders") OR (MH "Mental Disorders, Chronic") OR (MH "Depression") OR (MH "Generalized Anxiety Disorder") OR (MH "Obsessive-Compulsive Disorder") OR (MH "Panic Disorder") OR (MH "Stress Disorders, Post-Traumatic") OR (MH "Affective Disorders") OR (MH "Anxiety Disorders") OR (MH "Affective Disorders, Psychotic") OR (MH "Psychotic Disorders") OR (MH "Bipolar Disorder") OR (MH "Schizophrenia") OR (MH "Schizoaffective Disorder") OR (MH "Personality Disorders") OR (MH "Paraphilic Disorders") OR (MH "Neurotic Disorders")) OR (“mental illness” or “mental health disorder” or “mental problem” or “mental disorder” or “mental disease” or psychiatric or “psychotic disorder” or psychosis or psychotrauma or “mood disorder” or schizophrenia or “delusional disorder” or depressi* or “bipolar disorder” or anxiety or “panic disorder” or “obsessive-compulsive disorder” or “post-traumatic stress disorder” or “eating disorder” or “anorexia nervosa” or “bulimia nervosa” or “binge-eating disorder” or “personality disorder”).ti,ab.) AND (((MH "Ethnic Groups+") OR (MH "Minority Groups") OR (MH "Migrants") OR (MH "Immigrants") OR (MH "People of Color")) OR (ethnic* or racial* or race or cultur* or minority* or immigrant* or migrant* or native or Indigenous or Aboriginal or “First Nation*” or “African American” or “American Indian” or “Mexican American” or Black or White or Caucasian or European or Asian or Maori or Hispanic or Latino or Caribbean or “Middle Eastern” or Chinese).ti,ab.) AND (Publication date: 20140101-20241231)

**Scopus**

TITLE-ABS ( diabet* OR prediabet* OR "impaired glucose tolerance" OR "impaired fasting glucose" OR t1d* OR t2d* ) AND TITLE-ABS ( adult* OR "middle-aged" OR aged OR person* OR people OR patient* ) AND TITLE-ABS ( "mental illness" OR "mental health disorder" OR "mental problem" OR "mental disorder" OR "mental disease" OR psychiatric OR "psychotic disorder" OR psychosis OR psychotrauma OR "mood disorder" OR schizophrenia OR "delusional disorder" OR depressi* OR "bipolar disorder" OR anxiety OR "panic disorder" OR "obsessive-compulsive disorder" OR "post-traumatic stress disorder" OR "eating disorder" OR "anorexia nervosa" OR "bulimia nervosa" OR "binge-eating disorder" OR "personality disorder" ) AND TITLE-ABS ( ethnic* OR racial* OR race OR cultur* OR minority* OR immigrant* OR migrant* OR native OR indigenous OR aboriginal OR "First Nation*" OR "African American" OR "American Indian" OR "Mexican American" OR black OR white OR caucasian OR european OR asian OR maori OR hispanic OR latino OR caribbean OR "Middle Eastern" OR chinese ) AND PUBYEAR > 2013 AND PUBYEAR < 2025

Supplementary Figure 1.

Supplementary Figure 2.

In the figure, the vertical axis represents the log-transformed proportion of mental health disorders among individuals with diabetes, while the horizontal axis shows the year of publication. Each dark dot corresponds to a single study, with the size of the dot indicating the relative weight assigned to that study in the analysis. The slight downward slope of the regression line suggests a minor decline in the reported proportion of mental health disorders over time. However, this trend was not statistically significant (p = 0.159), indicating that the observed change may be due to random variation rather than a true temporal effect.

Appendix 2

**AXIS Checklist**

**NOS Criteria**
